# Impact of antimicrobial exposure at delivery and siblings on early *Bifidobacterium* succession and allergy development up to 24 months of age

**DOI:** 10.1186/s12866-025-04056-7

**Published:** 2025-05-28

**Authors:** Naruaki Imoto, Chie Kano, Hiroto Morita, Tatsuhiko Hirota, Fumitaka Amanuma, Hidekazu Maruyama, Shuko Nojiri, Shin Watanabe

**Affiliations:** 1 Advanced Research Institute for Health Science, Juntendo University, Bunkyo Ward, Tokyo, 113-8421 Japan; 2 Core Technology Laboratories, Asahi Quality & Innovations Ltd, Midori, Moriya, 302- 0106 Ibaraki Japan; 3 Department of Paediatrics, Department of Neonatology, Iwate Prefectural Iwai Hospital, Ichinoseki, 029-0192 Iwate Japan; 4 Clinical Research Support Centre, Juntendo University, Bunkyo Ward, Tokyo, 113-8421 Japan

**Keywords:** Allergy, Infant gut microbiota, Microbial diversity, Bifidobacterium, Bacteroides, Early-life factors, Weaning, Immune development

## Abstract

**Background:**

Allergic diseases such as asthma, eczema, and food allergies are rising globally. The infant gut microbiota, particularly the dominance of *Bifidobacterium*, shapes immune development and allergy risk. In Japan—where *Bifidobacterium* prevalence is notably high—longitudinal investigations focusing on the pre-weaning period, when external influences are relatively limited, remain scarce. Therefore, based on consistent hypotheses and findings from previous studies, we investigated how two important early factors—antibiotic exposure at birth and the presence of older siblings—influence the gut environment in early infancy and subsequent allergy development.

**Results:**

In a prospective cohort of 121 Japanese infants, stool samples were collected at seven time points from birth through 24 months. We quantified the relative abundances of *Bifidobacterium*, *Bacteroides*, *Clostridium*, and *Faecalibacterium* and recorded allergic outcomes at 2 years. Both antimicrobial exposure at delivery and sibling presence significantly altered gut microbiota composition and overall diversity in early infancy. Although the full cohort showed no consistent diversity or *Bifidobacterium* differences by allergic status, in several subgroups where these two factors were excluded, infants who had an allergy by 24 months exhibited marked shifts in early gut microbiota community structure—particularly in beta diversity—and reduced *Bifidobacterium* occupancy during the pre-weaning period (1–6 months) versus non-allergic peers. Moreover, infants whose gut microbiota was initially affected by these factors showed a recovery in diversity after weaning, a rebound more pronounced in non-allergic individuals.

**Conclusions:**

These findings indicate that both the initial community configuration and its capacity to rebound after perturbation are critical determinants of allergy risk. By focusing on dynamic changes through weaning and adjusting for decisive confounders, this study refines insight beyond prior cross-sectional work. Early interventions that preserve or restore microbial diversity and *Bifidobacterium* dominance may therefore offer a promising strategy to mitigate allergic disease development.

**Supplementary Information:**

The online version contains supplementary material available at 10.1186/s12866-025-04056-7.

## Background

The human microbiome encompasses bacteria, fungi, viruses, and other microorganisms inhabiting the skin surface, oral cavity, and gastrointestinal tract, with bacterial cells alone outnumbering human cells by approximately ten-fold. These microorganisms play crucial roles in maintaining health and influencing disease outcomes [1, 2]. The prevalence of allergic diseases such as asthma, eczema, and food allergies has rapidly increased, affecting over 20% of the population in developed countries [3–6]. Recent studies suggest that the composition and dynamics of the microbiome are associated with various diseases, including allergies [7–10].

The intestinal flora differs significantly based on factors such as race, environment, and life stage, with the most dynamic changes occurring during infancy [11–14]. Bacterial diversity evolves monthly during this early period, and by 2–3 years of age, the microbiota resembles that of an adult [15–18]. During these critical early years, the infant gut microbiota is shaped by genetic, environmental, and host-related factors, influencing susceptibility to a range of diseases, including allergies, obesity, and autoimmune disorders. These early microbial influences set the stage for long-term health trajectories [19]. Several perinatal factors, such as antibiotic use and the presence of older siblings, have been identified as major influencers of neonatal microbiota [7, 8, 20, 21]. The formation and evolution of these microbial communities are crucial for the development of the intestinal immune system, especially up to approximately 6 months of age when weaning begins. Disruptions in this period, termed “dysbiosis”, have been linked to an increased risk of various diseases later in life [22–28].

Despite extensive research, the precise role of specific bacteria, such as *Bifidobacterium*, and the overall balance of the microbiome in the development of allergic diseases remain unclear. There is no consensus regarding whether the presence of specific bacteria at key time points or the overall dynamics of the microbiome are predictors of allergy development. The timing of microbial effects, the factors influencing them, and their relationship with allergy development are not yet fully understood owing to varied research designs, highlighting the urgent need for longitudinal studies to map these interactions [9, 10, 24, 25, 28–30].

We conducted several studies based on the assumption that dynamic changes in the microbiota, rather than its static composition, are important for understanding the relationship between allergic diseases and specific factors. Consequently, we found that from early infancy up to the weaning period in Japanese infants, antimicrobial exposure at delivery (AED) and the presence of siblings have a significant impact on the *Bifidobacterium* genus, which is the most dominant genus in this population [31, 32]. The *Bifidobacterium* genus is known to maintain a relatively dominant state throughout the life of Japanese individuals [14, 33].

Based on consistent hypothesis setting and research design in previous studies, this study focused on factors that decisively influence the establishment of the intestinal environment in infancy, particularly the *Bifidobacterium* genus, which is characteristic to Japanese individuals, and several dominant bacteria that have previously been reported to be associated with the onset of allergic diseases. Hence, we investigated the temporal changes in these factors and their relationship with the onset of allergic diseases in children.

Specifically, we investigated the bacterial genera that are particularly dominant in Japanese infants, *Bifidobacterium* and *Bacteroides* [31, 32], which are known for their roles in maintaining intestinal immunity and suppressing inflammation through the production of short-chain fatty acids [14, 31, 32, 34, 35]. The main aim was to explore how chronological changes in these genera, along with the broader evolution of the microbiota, are related to the development of allergic diseases from birth to 2 years of age. Additionally, as a secondary objective, we investigated longitudinal changes in butyrate-producing bacteria, *Clostridium* and *Faecalibacterium*, which have been linked to the establishment of an infant gut environment and the prevention of allergies [24, 36]. Consequently, this study aimed to provide insight into early interventions that could prevent allergic diseases, potentially leading to significant public health benefits.

## Methods

### Participants

This prospective cohort study aimed to elucidate the relationship between temporal changes in intestinal flora in early infancy and the subsequent development of allergic diseases. In particular, we also examined how various factors that affect the intestinal environment in early infancy are involved in the development of allergic diseases.

This study was conducted in accordance with the Declaration of Helsinki and the Japanese “Ethical Guidelines for Medical and Health Research Involving Human Subjects.” This study was approved by the ethics committees of Juntendo University (approval number: 2017127) and Iwate Prefectural Iwai Hospital (approval number: 1234).

The participants included 142 infants and their mothers who consented to participate at Iwate Prefectural Iwai Hospital in Ichinoseki City, Iwate Prefecture, between February 2018 and March 2019, either before or within 2 weeks after delivery. These included infants born at term by vaginal delivery or caesarean section, and whose mothers had not received any antimicrobial agents from 1 month before delivery to the time of delivery, excluding the time of delivery. All mothers were provided explanations regarding the purpose and methods of the study, and written informed consent was obtained from all participants and their parents or guardians. Antimicrobials were administered at the time of delivery based on a protocol that had been established in advance at the Iwate Prefectural Iwai Hospital, with the details described in a previous report [31, 32]. 

The exclusion criteria included: preterm infants born before 37 weeks of age, infants who received oral or intravenous antibiotics before 6 months of age, and infants who could not be evaluated for allergic diseases up to 24 months of age because of a lack of questionnaires or samples after 12 months of age. Moreover, if the stool samples were unsuitable for analysis using MiSeq or if they were not submitted at a specific point in time, only the data at that point were treated as missing and excluded from the analysis.

### Stool sample collection and analysis

Stool samples were collected from the infants at 1, 3, 6, 9, 12, 18, and 24 months of age. For samples taken at 1, 3, and 6 months of age, the procedure was carried out following a previously reported method, whereby a sample collection kit was sent to the parents, and the samples were sent to the research facility after collection [32]. The procedure for collecting samples and sending them to the research facility from 9 months of age onwards was the same as that for 6 months of age.

DNA was extracted from the samples as described previously [32]. Briefly, the samples (2 mL) were transferred to plastic tubes, centrifuged at 14,000 × g for 3 min, washed in 1.0 mL of phosphate-buffered saline, and centrifuged at 14,000 × g. Pellets were re-suspended in 500 µL of extraction buffer (166 mM Tris/HCl, 66 mM EDTA, 8.3% sodium dodecyl sulphate, pH 9.0) and 500 µL of TE buffer-saturated phenol. Next, 300 mg of zirconium beads (0.1 mm diameter) were added to the suspension, and the mixture was vortexed vigorously three times for 60 s each using a Multi Beads Shocker (Yasui Kikai Corp., Osaka, Japan). After centrifugation at 14,000 × g for 5 min, 400 µL of the supernatant was purified using a Maxwell Instrument (Promega KK, Tokyo, Japan).

Sequencing of the 16 S rRNA gene of the processed samples was performed using a MiSeq V3 kit according to the manufacturer’s protocol (Illumina, CA, USA). Briefly, the V3-V4 region of the bacterial 16 S rDNA was amplified by PCR with forward and reverse primers (5′- TCG TCG GCA GCG TCA GAT GTG TAT AAG AGA CAG CCT ACG GGA GGC WGC AG-3′ and 5′-GTC TCG TGG GCT CGG AGA TGT GTA TAA GAG ACA GGA CTA CHV GGG TAT CTA ATC C-3′), using the TaKaRa Ex Taq HS Kit (TaKaRa Bio, Shiga, Japan) to amplify 5 ng of DNA from a faecal sample. After the PCR products were purified using Agencourt AMPure XP (Beckman Coulter, CA, USA), they were amplified using the Nextera XT Index Kit v2 (Illumina, CA, USA). After the second round of PCR, the products were purified using Agencourt AMPure XP. The library was quantified, normalised, and pooled in equimolar amounts. Sequencing was conducted using a paired-end 2 × 300-bp cycle run on an Illumina MiSeq system with MiSeq Reagent Kit v.3 (600 cycles).

All subsequent data processing and analyses were performed using QIIME2 (Quantitative Insights into Microbial Ecology, http://qiime2.org/) version 2021.2 [37]. The quality of the sequences was checked and filtered using the QIIME2 plugin DADA2 [38], and chimeric sequences were removed. The primers were trimmed and the remaining forward and reverse sequences were truncated to a final length of 280 bp. Taxonomic classification was performed using the feature-classifier plugin with a classifier based on the SILVA (version 138.1) genomic database. Alpha and beta diversities were analysed using Qiime2 plugins. Alpha diversity was analysed using Shannon and chao1 indexes, whereas beta diversity was assessed using UniFrac distances in the unweighted and weighted versions.

### Data collection

Regarding data collection, we used the Iwate Prefectural Iwai Hospital medical records and questionnaires for parents to obtain information about the infants and their mothers who were participants in the study. For each infant, we collected data on age, sex, birth and consultation weights, height, head circumference, abdominal circumference, underlying diseases, perinatal history, medication history (especially regarding antibiotic use), vaccination history, family history, lifestyle history, method of nutrition intake (breast milk or formula), meal content, stool characteristics, growth status, presence or absence of allergic diseases, and presence or absence of other diseases (such as infectious diseases) from 1 to 24 months of age. For mothers, we collected information on age, height and weight (body mass index calculation), history of childbirth, history of pre-existing conditions, history of allergies (food allergies, bronchial asthma, atopic dermatitis, and allergic rhinitis), abnormal findings at the time of delivery, use of antibiotics in the third trimester of pregnancy, systemic antibiotics administered during delivery (including type), family history (including information on the infants siblings), lifestyle history, medical history, stool characteristics, and history of taking supplements.

Questionnaires were sent to the parents along with the sample collection containers at the time of stool sample collection and, after completion, were returned to the research facility along with the samples (see the English translation of the allergy questionnaire in Additional File 1). No samples were collected at 15 and 21 months of age, and only the allergy questionnaire was sent to the parents, which was completed and returned to the Asahi Group Holdings (Sagamihara City, Kanagawa Prefecture) research facility.

### Diagnosis and types of allergy

Although the diagnosis of allergic diseases is often based on routine medical examinations, there is a possibility of subjective bias if the diagnosis is based solely on medical records and parental questionnaires. Therefore, in this study, an allergy diagnosis was confirmed by paediatric specialists certified by the Japanese Society of Paediatrics and the Japan Medical Specialty Board using domestic and international diagnostic criteria.

Infants and young children with recurrent wheezing or the possibility of future asthma development were evaluated using the modified Asthma Predictive Index (mAPI) [39], and those with four or more episodes of sleep disorders and wheezing at least four times a year met at least one of the main items (history of asthma in parents, diagnosis of atopic dermatitis, and response to inhaled allergens) or at least two of the secondary items (food allergy, increased eosinophil counts, and wheezing not associated with a cold).

For atopic dermatitis, the UK Working Party’s diagnostic criteria [40] was used. The main criteria for diagnosis were the presence of itchiness of the skin or itchiness reported by the parents, and the diagnosis was made when three or more of the following five items were met: presence of itchy skin symptoms on the inside of the elbows, behind the knees, in front of the ankles, and around the neck (including the cheeks in children aged 9 years and less); a history of asthma or hay fever, or a history of allergic disease in a first-degree relative; general skin dryness in the past 12 months; eczema on the inside of the joints (in children under 3 years, this included the cheeks, forehead, and outer sides of the limbs); and having developed the condition at 1 year of age or younger (this criterion was not used for children under 3 years of age).

The diagnosis of food allergy was made based on the “Guidelines for the Diagnosis and Treatment of Food Allergy” by the Japanese Ministry of Health, Labour and Welfare Research Group [41] and National Institute for Health and Care Excellence clinical guidelines [42]. Blood tests for food allergies were recommended in cases where: there was a history of immediate allergic symptoms; atopic dermatitis was diagnosed and there was little improvement despite appropriate skin care and treatment; no other causes, such as infection, had been identified; and the skin symptoms (pruritus, erythema, facial), digestive symptoms (oral itching, vomiting, diarrhoea, abdominal pain, constipation, growth disorders, refusal to eat or dislike certain foods), respiratory symptoms (upper respiratory symptoms, lower respiratory symptoms), and anaphylactic symptoms developed within a few minutes to a few hours after ingestion of a suspected substance. Infants and young children who were tested at the request of their parents or at the direction of their childcare facility, and who tested positive for blood antigen-specific IgE antibodies at a level of 0.3 IU/ml or higher, were identified from medical records.

In the present study, the diagnosis of allergic diseases was made based on medical records at 24 months of age, in addition to allergy questionnaires sent at 15 and 21 months of age.

### Statistical analysis

To analyse the differences between the allergic and non-allergic groups, the Kruskal-Wallis test was used for alpha diversity and permutational multivariate analysis of variance (PERMANOVA) for beta diversity. Dot and box plots for the diversity analysis were created using the ggplot2 package in R (X64 version 4.0.2). QIIME2 was used to analyse 16SrRNA, and SAS software version 9.4 (SAS Institute Inc., Cary, NC, USA) was used to compare the abundance of each genus calculated by QIIME2 and to analyse temporal changes. The association between background factors and the incidence of allergic diseases up to 24 months of age were assessed using logistic regression analysis. A logistic regression model was employed to examine the impact of these background factors on the relative abundance of key bacterial genera at each age. Based on the results, we created a volcano plot using R to observe the influence of background factors on each bacterial genus. Differences in bacterial genus abundance between the allergy-onset and non-onset groups were evaluated using the Mann–Whitney U test. Chronological changes in the dominant bacterial genera were analysed using a linear mixed-effects model incorporating random intercepts and a first-order autoregressive structure to account for repeated measures. Continuous variables related to maternal and infant background factors were compared using the Mann–Whitney U test, whereas categorical variables were analysed using Pearson’s chi-squared test. Statistical significance was set at a p-value of less than 0.05.

## Results

### Study population

Of the 142 participants who provided consent, three premature infants, 14 who received antimicrobial treatment before 6 months of age, and four with missing data after 12 months were excluded. Hence, a total of 121 participants were analysed. Stool samples analysed at each age were as follows: 119 at 1 month; 120 at 3, 6, and 9 months; 116 at 12 and 18 months; and 113 at 24 months, for a total of 824 samples.

Medical records and questionnaire results showed that all infants were weaned at 6 months of age.

### Background factors

Among the 121 infants analysed, 52 (43%) were diagnosed with allergic diseases, 41 (33.9%) with atopic dermatitis, 21 (17.4%) with recurrent wheezing, and 12 (9.9%) with food allergies (Table 1). In the allergy group, 36.5% of infants had two or more allergy phenotypes.

**Table 1:** Background factors of infants and their mothers

Background factors		Data
Number of infants		121
Number of girls		70 (57.9%)
Gestational age at birth		275.4 ± 9.0
Birth weight		3052 ± 335.9
Diagnosis of allergy		52(43.0%)
	Atopic dermatitis	41(33.9%)
	Recurrent wheezing	21(17.4%)
	Food allergy	12(9.9%)
Maternal antimicrobial use at delivery		66(54.5%)
Caesarean section		25(20.7%)
Antimicrobial exposure after birth from 6 m.o.		64(52.9%)
Antimicrobial exposure after birth from 6 m.o. to 12 m.o.		35(28.9%)
Infants with older siblings		62(51.2%)
Exclusive breast feeding		77(63.6%)
Age of mothers		31.6 ± 5.0
Maternal history of allergy		52(43.0%)

For each factor, the number of each category variable and the percentage it represents of the total are displayed as percentages, and for continuous variables, the mean and standard deviation are indicated

Among the total population, 28.9% of infants received antimicrobial agents between 6 and 12 months of age, whereas 52.9% received them between 6 and 24 months of age. However, for children who received antimicrobials from 6 to 12 months of age, there were no differences in background factors in the development of allergy in either the total population or subgroups (Table 1, Supplementary Tables 1–3).

A comparison of the background factors between the allergic and non-allergic groups, including subgroups such as exposure to antibiotics during delivery, presence or absence of siblings, and mode of delivery, revealed no significant differences (Supplementary Tables 1 and 2). Furthermore, no significant differences in background factors were found within each allergic phenotype (Supplementary Table 3).

### Proportion of Bacterial Genera Over Time

**Fig. 1:**
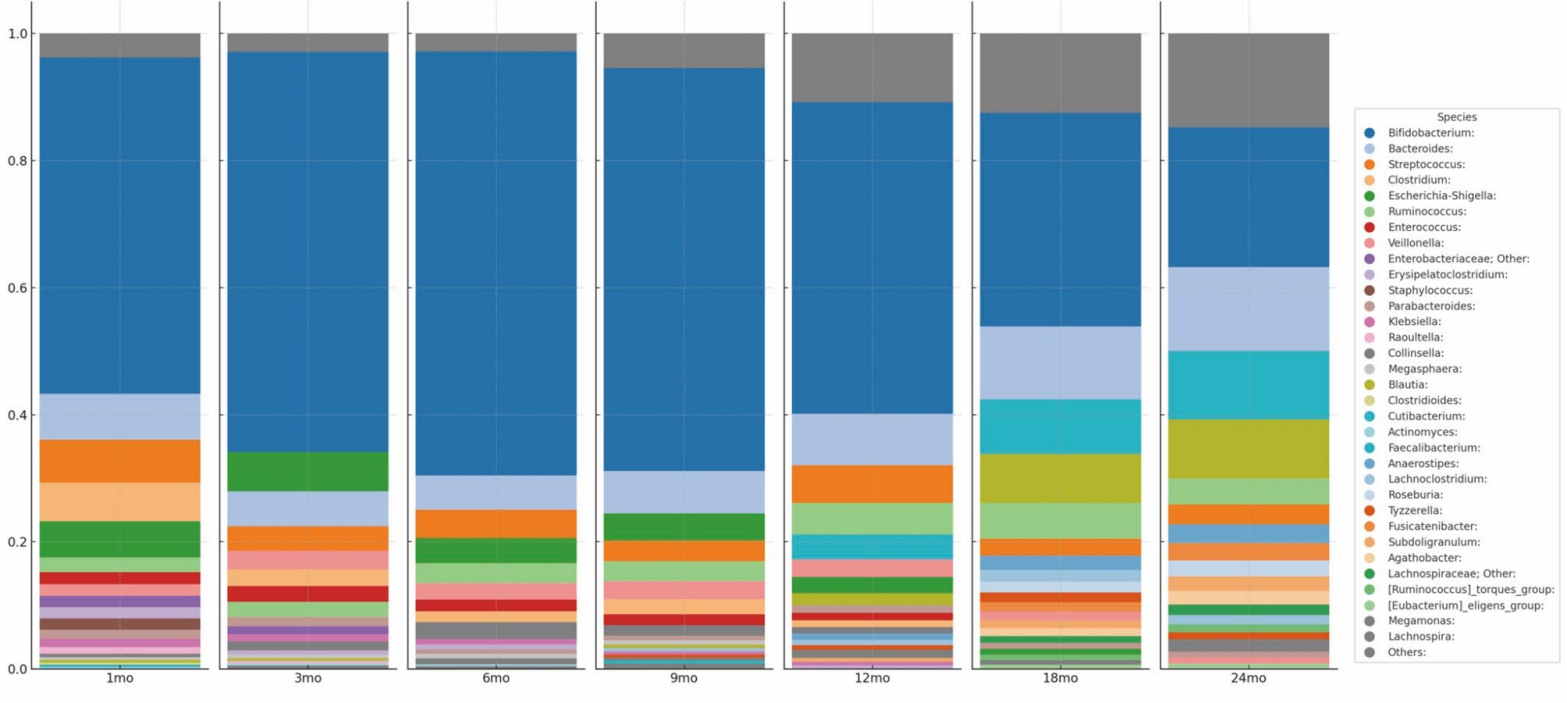
Top 20 bacterial genera in the gut microbiota of infants according to age. The bar graph shows the relative values (means) of the top 20 bacterial genera in the intestinal environment of infants in each age group. “Others” includes bacterial genera that did not make the top 20

At all ages, *Bifidobacterium* was the most dominant genus, followed by *Bacteroides*, except at 3 months of age when *Bacteroides* was the third most dominant genus. The relative abundance of *Bifidobacterium* peaked at 6 months and decreased thereafter, with a variety of bacterial genera present from 12 months onwards. *Clostridium* was the fourth most dominant genus at 1 month of age, and remained in the top 10 until approximately 9 months, after which its abundance decreased. In contrast, *Faecalibacterium* had a relatively low abundance up to 9 months of age, but became one of the top five genera from 12 months onwards, with its abundance increasing over time (Fig. 1).

### Relationship between allergies and background factors

In the total cohort, there was no significant relationship between the diagnosis of allergic diseases by 24 months of age and major background factors, including antimicrobial exposure after 6 months of age (Table 2). However, in a subgroup analysis of vaginally delivered infants, infants of mothers without a history of allergies were less likely to develop allergies (odds ratio [OR]: 0.337; 95% confidence interval [CI]: 0.127–0.892) (Supplementary Table 4).

**Table 2:** Influence of each background factor on the development of allergies up to 24 months of age

Background factors	Odds Ratios	95%CI	
Gestational days	0.987	0.928	1.05
Birth weight	1	0.999	1.001
Age of mothers	0.956	0.884	1.035
Female	0.797	0.363	1.75
Vaginal delivery	1.921	0.492	7.499
Infants without siblings	0.586	0.256	1.341
Non-AED	0.723	0.303	1.723
Mothers without allergy	0.563	0.251	1.265
Exclusively breast-fed	0.814	0.363	1.826
Without antimicrobial exposure after 6 m.o.	0.601	0.232	1.559
Without antimicrobial exposure after 6 m.o. to 12 m.o.	1.429	0.497	4.105

The odds ratio and 95% confidence interval were calculated using logistic regression analysis. The significance level was set at 5%.　AED means infants group with antimicrobial exposure at delivery

### Bacterial genera and background factors

**Fig. 2:**
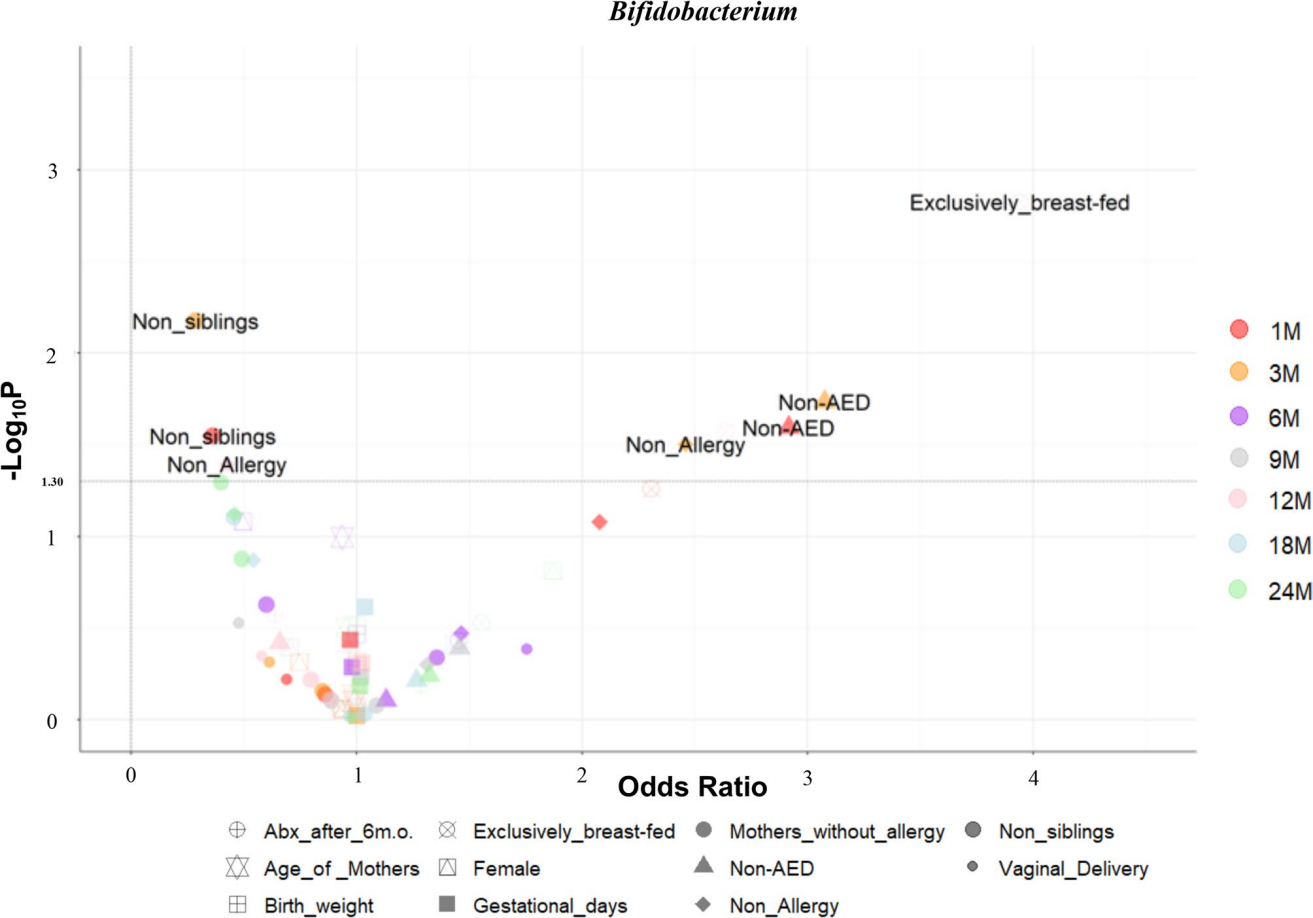
Background factors affecting the relative abundance of *Bifidobacterium*. To visualise the influence of background factors on the relative abundance of *Bifidobacterium*, two values representing low and high relative abundances were selected based on the median, and a volcano plot was created with the odds ratio for high relative abundance on the x-axis and the logarithm of the p-value (-Log_10_P) on the y-axis. The value 1.30 on the Y-axis is used as the threshold value, and plots above this value (*p* < 0.05) are labelled with the names of each factor. Each factor is colour-coded according to the age of the subject. Abx_after_6m.o. indicates exposure to antibiotics after 6 months of age, and AED denotes antimicrobial exposure at delivery

At 1 month of age, the relative abundance of *Bifidobacterium* was significantly associated with the presence or absence of siblings (OR: 0.336; 95% CI: 0.14–0.797) and AED (OR: 2.89; 95% CI: 1.14–7.349) **(**Fig. 2, Supplementary Fig. 1). *Bacteroides* was significantly associated with the mode of delivery (OR: 13.11; 95% CI: 3.19–53.94). Similarly, *Clostridium* was associated with the mode of delivery, siblings, AED, and feeding methods. These associations persisted at 3 months of age, where *Bifidobacterium* was also linked to the presence or absence of allergies by 24 months of age (OR: 2.386; 95% CI: 1.07–5.329). At 6 months of age, the relative abundance of *Clostridium* was significantly associated with the feeding method; however, there was no association between the relative abundance of *Bifidobacterium* or *Bacteroides* and background factors. At 9 months, *Bifidobacterium* was associated with the feeding method, whereas *Bacteroides* and *Clostridium* were associated with the mode of delivery. At 12 months of age, *Bifidobacterium* was significantly associated with the feeding method and allergy status by 24 months, whereas *Bacteroides* was associated with the feeding method, allergy status, sex, and mode of delivery. *Clostridium* was associated with AED at 12 months of age; however, no associations were found with *Faecalibacterium* at any age.

No significant effect on any of the genera was observed for children who received antimicrobials after 6 months of age and those who received antimicrobials before 12 months of age (Supplementary Table 5).

### Comparison of the intestinal environment between infants with and without allergy up to 24 months of age

#### General analysis

**Fig. 3:**
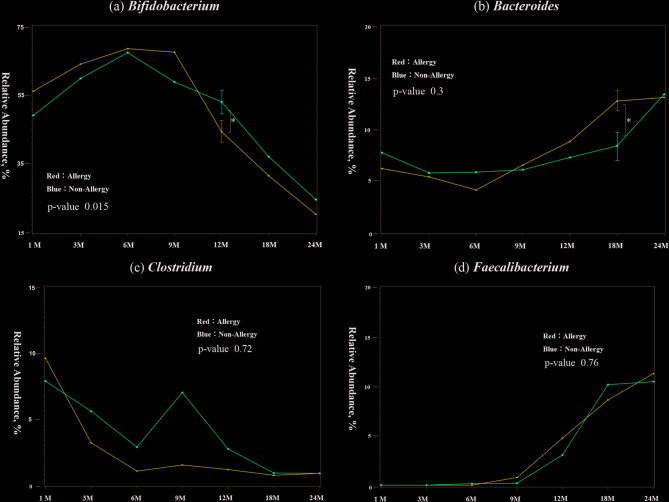
Bacterial genus occupancy in infants with or without allergies by 24 months of age (*N* = 121) In each graph for each bacterial genus, the vertical axis shows the relative abundance in the intestinal environment, and the horizontal axis shows the seven time points from birth to 24 months of age (1, 3, 6, 9, 12, 18, and 24 months). The comparison of changes in the occupancy rate over time using a linear mixed-effects model is shown by the red line for the allergic group (that developed allergies by 24 months of age) and the blue line for the non-allergic group, and the p-value is shown as the result of the comparison. Additionally, the Mann–Whitney U test was used for the comparison of occupancy rates at each time point, the standard error is shown as a bar for those that showed a significant difference.　The significance level was set at 5%. *p < 0.05, **p < 0.01, ***p < 0.001

There were no significant differences in alpha and beta diversity between the allergic and non-allergic groups at any age (Fig. 3). However, *Bifidobacterium* was significantly more abundant in the allergic group at 12 months (*p* = 0.04), whereas *Bacteroides* was more abundant in the non-allergic group at 18 months (*p* = 0.02). Additionally, a significant difference in *Bifidobacterium* changes over time were observed between the two groups (*p* = 0.015). *Clostridium* and *Faecalibacterium* showed no significant differences, although *Clostridium* decreased in abundance in the non-allergic group around weaning and increased in the allergic group after 9 months of age. *Faecalibacterium* was low in both groups until weaning but subsequently increased.

#### Subgroup analysis

**Fig. 4:**
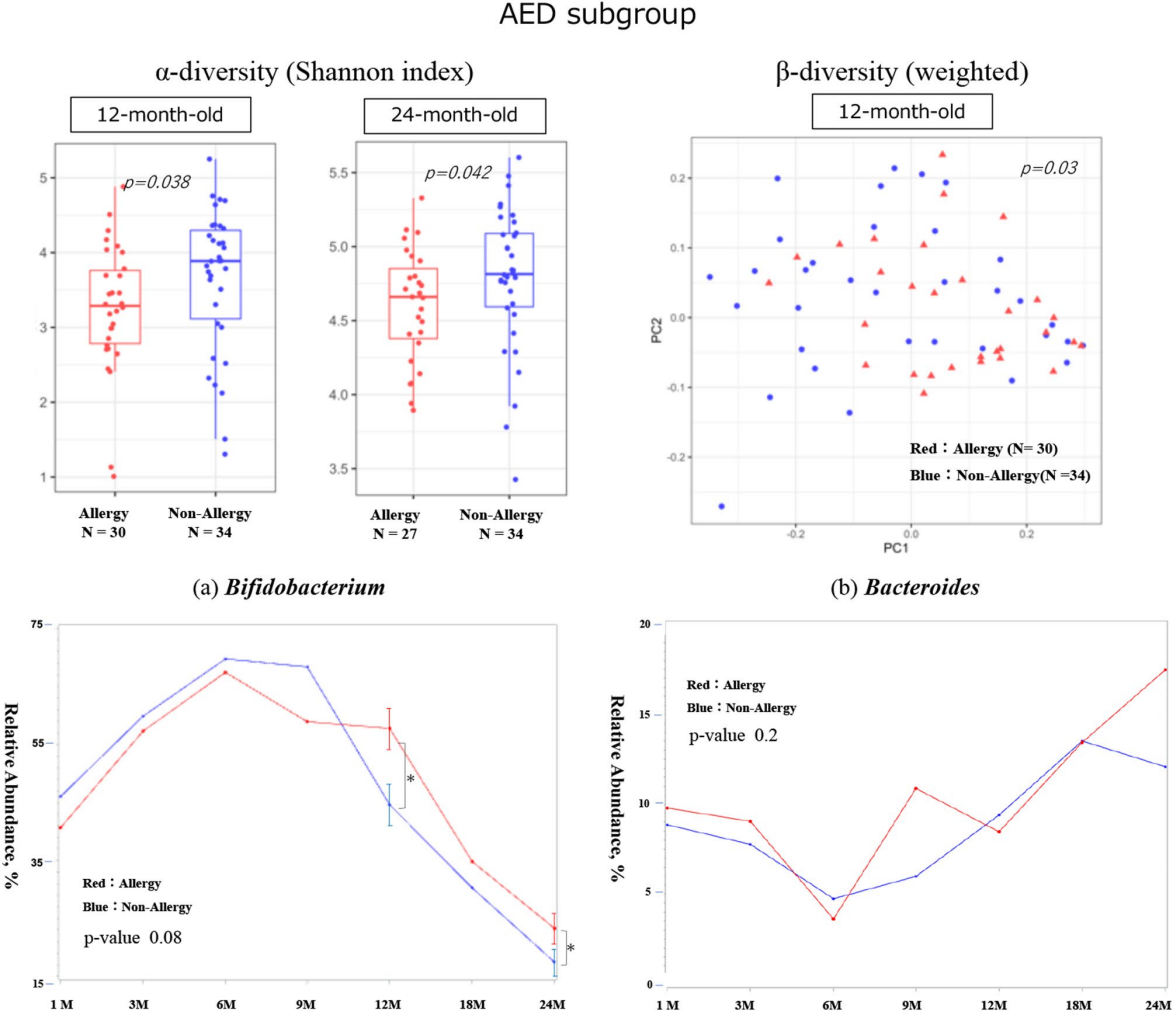
Gut diversity and genus occupancy in AED-exposed infants with or without allergies by 24 months of age (*N* = 66). The results of the diversity analysis are only shown for the months in which a significant difference was found, and a p-value indicates each result. In each graph for each bacterial genus, the vertical axis shows the relative abundance in the intestinal environment, and the horizontal axis shows the seven time points from birth to 24 months of age (1, 3, 6, 9, 12, 18, and 24 months). The comparison of changes in the occupancy rate over time using a linear mixed-effects model is shown by the red line for the allergic group (that developed allergies by 24 months of age) and the blue line for the non-allergic group, and the p-value is shown as the result of the comparison. Additionally, the Mann–Whitney U test was used for the comparison of occupancy rates at each time point, the standard error is shown as a bar for those that showed a significant difference.　The significance level was set at 5%. *p < 0.05, **p < 0.01, ***p < 0.001.　AED, antimicrobial exposure at delivery

In the subgroup of infants who received antibiotics at birth (AED subgroup), significant differences in alpha diversity were observed at 12 months of age, with Chao1 (*p* = 0.045) and Shannon diversity (*p* = 0.038) being higher in the non-allergic group. At 24 months of age, the Shannon diversity index remained significantly higher in the non-allergic group (*p* = 0.042). Beta diversity also showed a significant difference at 12 months of age (*p* = 0.03). Regarding the relative abundance of *Bifidobacterium*, no differences were observed between the allergic and non-allergic groups during the early infant period (up to 6 months of age); however, at 12 and 24 months, *Bifidobacterium* was more abundant in the allergic group (*p* = 0.02 at both ages) (Fig. 4). There were no significant differences between the groups in terms of occupancy at each month or changes over time for *Clostridium* or *Faecalibacterium* (Supplementary Fig. 2).

Regarding infants who did not receive antibiotics at birth (non-AED subgroup), there were no significant differences in the diversity analysis (Supplementary Table 6), relative abundance of *Bifidobacterium* and *Bacteroides* at each age, or changes over time. Although no significant differences were found in the relative abundance of *Clostridium* by age, a significant difference was observed in changes over time; however, *Faecalibacterium* showed no significant difference (Supplementary Fig. 3).

**Fig. 5:**
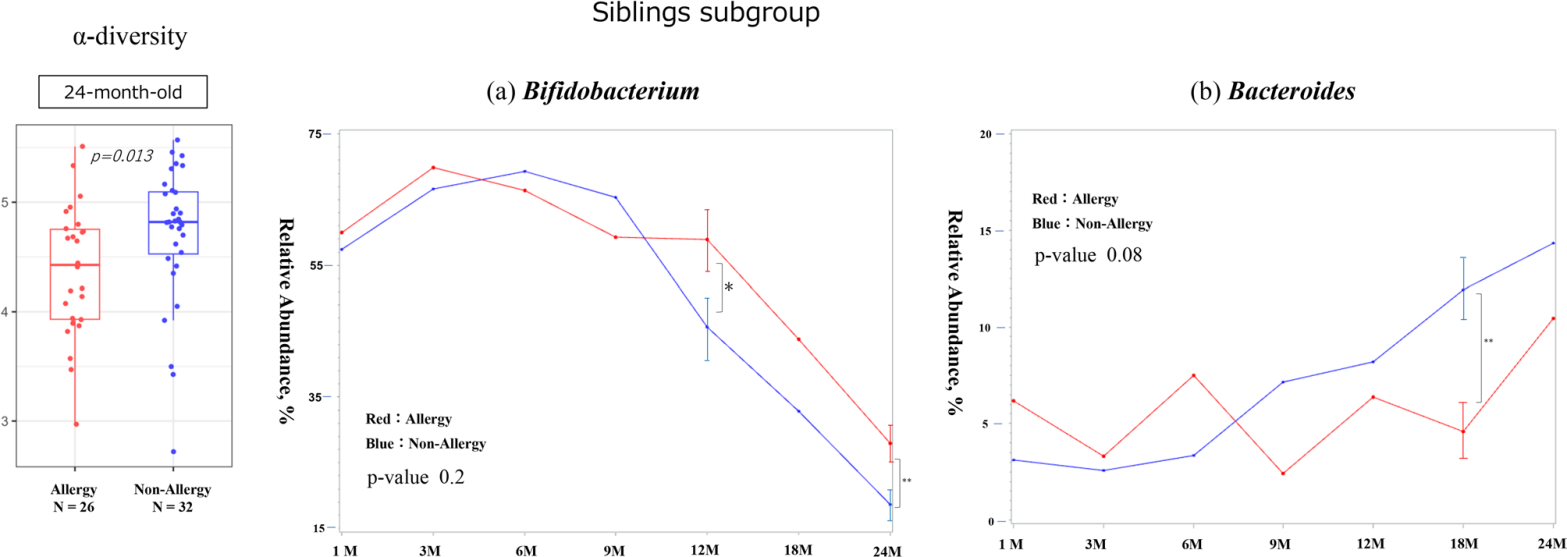
Gut diversity and genus occupancy in sibling-exposed infants with or without allergies by 24 months of age (*N* = 62). The results of the diversity analysis are only shown for the months in which a significant difference was found, and a p-value indicates each result. In each graph for each bacterial genus, the vertical axis shows the relative abundance in the intestinal environment, and the horizontal axis shows the seven time points from birth to 24 months of age (1, 3, 6, 9, 12, 18, and 24 months). The comparison of changes in the occupancy rate over time using a linear mixed-effects model is shown by the red line for the allergic group (that developed allergies by 24 months of age) and the blue line for the non-allergic group, and the p-value is shown as the result of the comparison. Additionally, the Mann–Whitney U test was used for the comparison of occupancy rates at each time point, the standard error is shown as a bar for those that showed a significant difference.　The significance level was set at 5%. *p < 0.05, **p < 0.01, ***p < 0.001

In the subgroup of infants with older siblings (sibling subgroup), a significant difference in alpha diversity was noted at 24 months (Shannon index, *p* = 0.013), with non-allergic infants showing higher diversity. No significant differences in the relative abundances of *Bifidobacterium* and *Bacteroides* were observed up to 6 months of age. However, for *Bifidobacterium*, the allergic group exhibited a significantly higher relative abundance at 12 and 24 months of age (*p* = 0.04 and 0.009, respectively). For *Bacteroides*, the non-allergic group showed a significantly higher relative abundance of *Bacteroides* at 18 months of age (*p* = 0.005) (Fig. 5); however, no significant differences were observed in *Clostridium* or *Faecalibacterium* at any age (Supplementary Fig. 4).

**Fig. 6:**
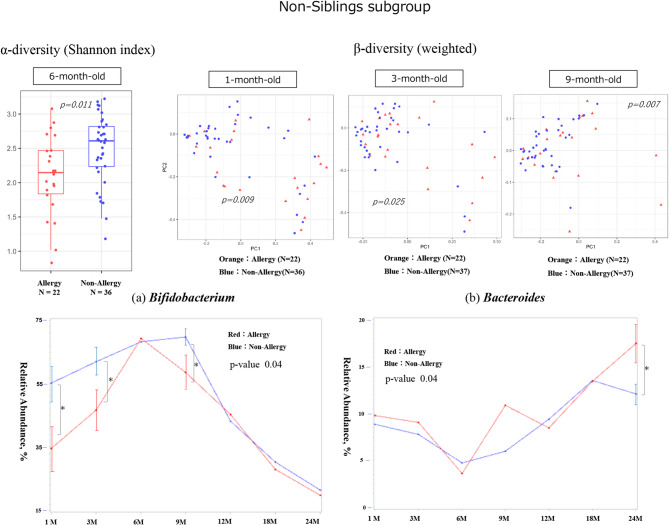
Gut diversity and genus occupancy in sibling-free infants with or without allergies by 24 months of age (*N* = 59). The results of the diversity analysis are only shown for the months in which a significant difference was found, and a p-value indicates each result. In each graph for each bacterial genus, the vertical axis shows the relative abundance in the intestinal environment, and the horizontal axis shows the seven time points from birth to 24 months of age (1, 3, 6, 9, 12, 18, and 24 months). The comparison of changes in the occupancy rate over time using a linear mixed-effects model is shown by the red line for the allergic group (that developed allergies by 24 months of age) and the blue line for the non-allergic group, and the p-value is shown as the result of the comparison. Additionally, the Mann–Whitney U test was used for the comparison of occupancy rates at each time point, the standard error is shown as a bar for those that showed a significant difference.　The significance level was set at 5%. *p < 0.05, **p < 0.01, ***p < 0.001

In infants without older siblings (non-sibling subgroup), a significant difference in alpha diversity was observed at 6 months (Shannon, *p* = 0.011). Beta diversity also showed significant differences at 1, 3, and 9 months (*p* = 0.009, 0.025, and 0.007, respectively). The relative abundance of *Bifidobacterium* was significantly lower in the allergic group at 1, 3, and 9 months (*p* = 0.01, 0.03, and 0.02, respectively). There was also a significant difference in the changes over time (*p* = 0.04). *Bacteroides* was significantly higher in the allergic group at 24 months of age (*p* = 0.04) (Fig. 6). *Clostridium* showed no significant differences in relative abundance by age but exhibited significant differences in changes over time. However, *Faecalibacterium* showed no significant difference in any comparison (Supplementary Fig. 5).

**Fig. 7:**
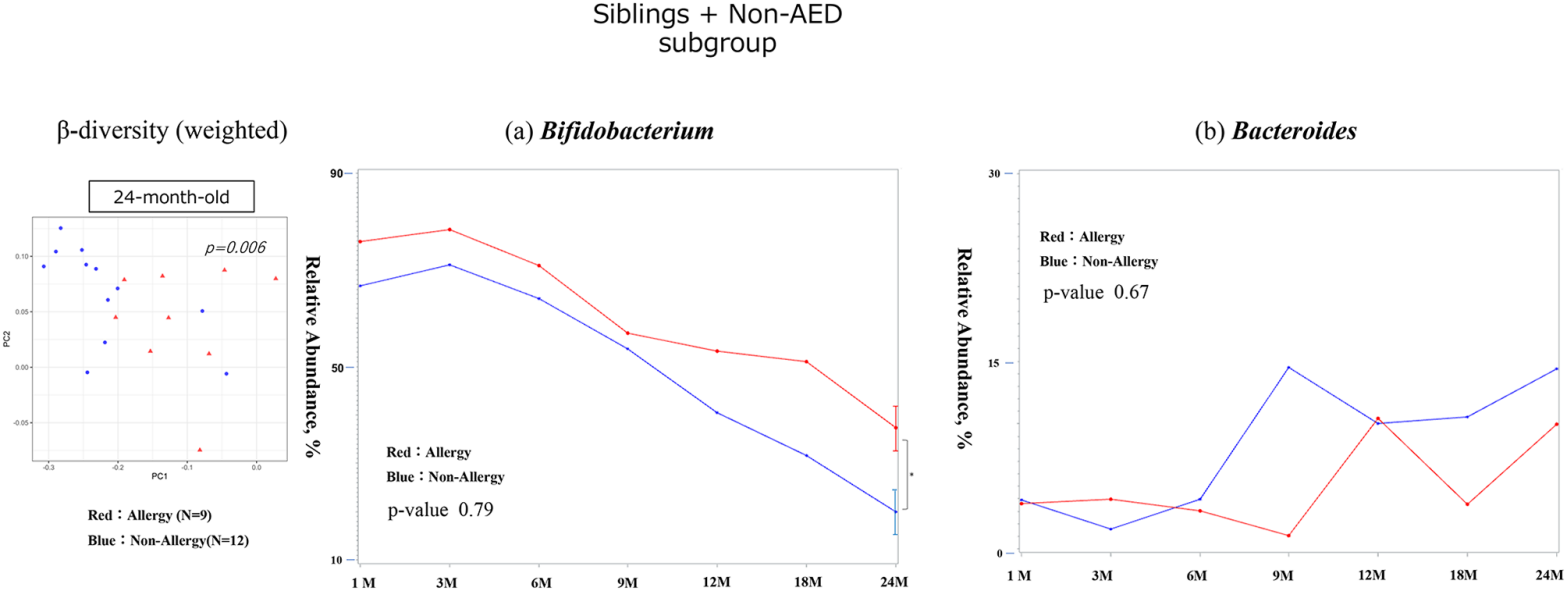
Gut diversity and genus occupancy in sibling-exposed, non-AED infants with or without allergies by 24 months of age (*N* = 22). The results of the diversity analysis are only shown for the months in which a significant difference was found, and a p-value indicates each result. In each graph for each bacterial genus, the vertical axis shows the relative abundance in the intestinal environment, and the horizontal axis shows the seven time points from birth to 24 months of age (1, 3, 6, 9, 12, 18, and 24 months). The comparison of changes in the occupancy rate over time using a linear mixed-effects model is shown by the red line for the allergic group (that developed allergies by 24 months of age) and the blue line for the non-allergic group, and the p-value is shown as the result of the comparison. Additionally, the Mann–Whitney U test was used for the comparison of occupancy rates at each time point, the standard error is shown as a bar for those that showed a significant difference.　The significance level was set at 5%. *p < 0.05, **p < 0.01, ***p < 0.001.　AED, antimicrobial exposure at delivery

Among the infants with older siblings and who did not receive antibiotics at birth (siblings + non-AED subgroup), a significant difference in beta diversity was observed at 24 months of age (*p* = 0.006). The allergic group had a significantly higher *Bifidobacterium* abundance at 24 months (*p* = 0.01), although the allergic and non-allergic groups showed high *Bifidobacterium* abundance during early infancy. There were no significant differences in *Bacteroides* or changes over time in either genera (Fig. 7). *Faecalibacterium* was more abundant in the non-allergic group at 24 months, with no significant difference in *Clostridium* (Supplementary Fig. 6).

**Fig. 8:**
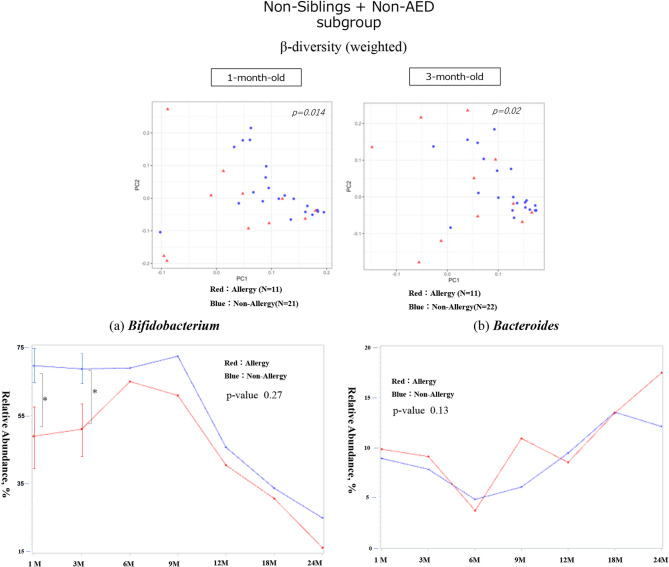
Gut diversity and genus occupancy in non-sibling, non-AED infants with or without allergies by 24 months of age (*N* = 33) The results of the diversity analysis are only shown for the months in which a significant difference was found, and a p-value indicates each result. In each graph for each bacterial genus, the vertical axis shows the relative abundance in the intestinal environment, and the horizontal axis shows the seven time points from birth to 24 months of age (1, 3, 6, 9, 12, 18, and 24 months). The comparison of changes in the occupancy rate over time using a linear mixed-effects model is shown by the red line for the allergic group (that developed allergies by 24 months of age) and the blue line for the non-allergic group, and the p-value is shown as the result of the comparison. Additionally, the Mann–Whitney U test was used for the comparison of occupancy rates at each time point, the standard error is shown as a bar for those that showed a significant difference.　The significance level was set at 5%. *p < 0.05, **p < 0.01, ***p < 0.001.　AED, antimicrobial exposure at delivery

In infants without siblings and who received antibiotics at birth (non-siblings + AED subgroup), no significant differences were found in the diversity analysis (Supplementary Table 6), in the relative abundance of bacterial genera at each age, or in changes over time (Supplementary Fig. 7). The relative abundance of *Bifidobacterium* in this subgroup was lower than that in other subgroups.

In infants without siblings and who did not receive antibiotics at birth (non-sibling + non-AED subgroup), beta diversity was significantly different at 1 and 3 months of age (*p* = 0.014 and 0.024, respectively). At 1 and 3 months of age, *Bifidobacterium* was significantly lower in the allergic group (*p* = 0.04 and 0.03, respectively). No significant differences were observed for *Bacteroides* or changes over time in either genus (Fig. 8). Moreover, *Clostridium* and *Faecalibacterium* showed no significant differences in any of the comparisons (Supplementary Fig. 8).

In the subgroup analysis based on the delivery method, infants born vaginally showed no significant difference in diversity between the groups; however, at 9 months of age, *Bifidobacterium* was significantly lower in the allergic group (*p* = 0.04). No significant differences were observed with the Caesarean section group (Supplementary Figs. 9 and 10). Moreover, no significant differences were found in *Bacteroides*, *Clostridium*, or *Faecalibacterium* in either of the subgroups.

### Summary of changes in *Bifidobacterium* over time

**Fig. 9:**
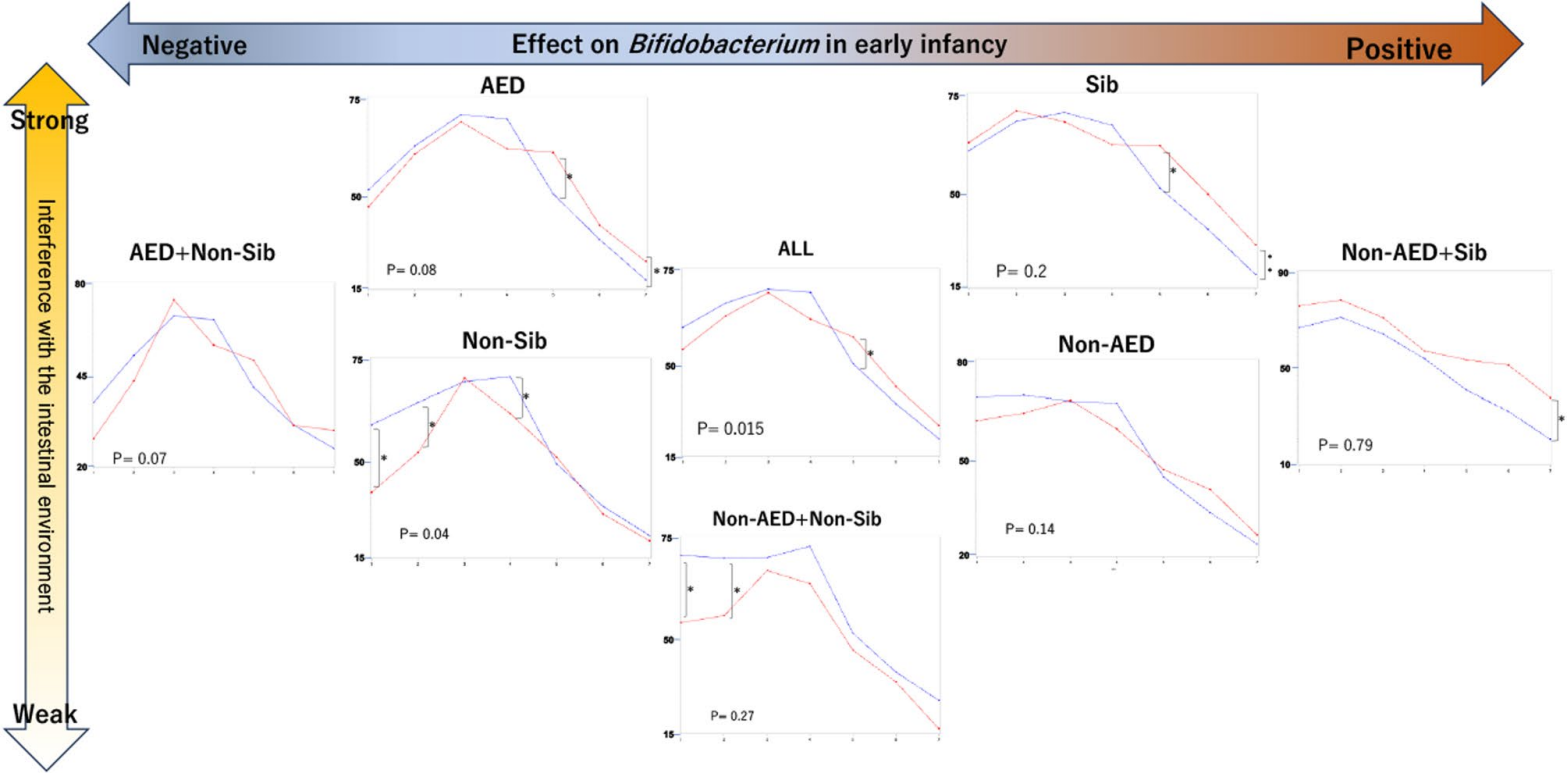
*Bifidobacterium* occupancy changes over 24 months with and without allergies, depending on the effect and interference on the gut. ALL indicates the entire study population; AED and Non-AED indicate infants with and without antimicrobial exposure at delivery, respectively; Sib and Non-Sib indicate infants with and without older siblings, respectively; In the graphs for each group, the vertical axis shows the relative abundance of *Bifidobacterium* in the intestinal environment, and the horizontal axis shows the age of the infant (1, 3, 6, 9, 12, 18, and 24 months) from youngest to oldest, numbered from 1 to 7. The red line represents the relative values of the allergy group, and the blue line represents the relative values of the non-allergy group, showing the change over time. The p-value in the graph shows the results of comparing the change over time in the two groups using a linear mixed effects model. The results of the comparison of the proportion of each group at each age (Mann–Whitney’s U test) are also shown in each graph for those that showed a significant difference. The significance level was set at less than 5%. *p < 0.05, ** *p* < 0.01.a

Changes in the relative abundance of *Bifidobacterium* over time were influenced by AED and the presence of siblings. Infants with higher early *Bifidobacterium* levels tended to maintain a higher abundance in both groups. When interference (positive or negative) was more pronounced, the allergic group showed higher *Bifidobacterium* levels than the non-allergic group after 12 months of age, whereas lower interference resulted in higher *Bifidobacterium* levels in the non-allergic group during early infancy (Fig. 9).

### Phenotype analysis results

For atopic dermatitis, no relationship was found with background factors; however, having siblings was associated with a lower risk for the development of recurrent wheezing (OR: 0.29; 95% CI: 0.08–0.98) and food allergy (OR: 0.14; 95% CI: 0.02–0.92). In cases of food allergies, antibiotic administration after 6 months of age was associated with a higher risk (OR: 0.06; 95% CI: 0.007–0.51) (Supplementary Table 7).

There were no significant differences in the background factors between infants with and without any type of allergy up to 24 months of age (Supplementary Table 8). At 12 months of age, the relative abundance of *Bacteroides* was associated with recurrent wheezing (OR: 3.96; 95% CI: 1.15–13.7) in the non-wheezing group, whereas *Bifidobacterium* at 24 months of age was associated with atopic dermatitis (OR: 0.25; 95% CI: 0.09–0.70) in the non-dermatitis group (Supplementary Table 9).

### Diversity and bacterial comparisons by phenotype

In atopic dermatitis, significant differences in beta diversity (weighted) were observed at 9 months of age (*p* = 0.02). For recurrent wheezing, significant differences in alpha diversity (Shannon index) were seen at 6 months of age (*p* = 0.03) and in beta diversity (weighted) at 18 months of age (*p* = 0.03). For food allergies, significant differences in alpha diversity (Shannon) were noted at 6 and 9 months of age (*p* = 0.03 both) (Supplementary Table 10).

A summary of the changes in *Bifidobacterium* based on AED and siblings showed a similar trend in the atopic dermatitis and allergic groups. For recurrent wheezing, there were not enough cases in some subgroups to determine a clear trend; however, a significant difference in *Bifidobacterium* relative abundance was observed at 12 months of age between the allergic and non-allergic groups in infants who received antibiotics at birth. Regarding food allergies, the small sample size (12 cases) limited comparisons, and no clear differences in the relative abundance or changes over time were found between the groups at each age (Supplementary Fig. 11).

## DIscussion

The findings of this study demonstrated that the presence of siblings and AED significantly affected the gut microbiota in early infancy, particularly influencing the dominant genus *Bifidobacterium*, with siblings having a positive impact and AED having a negative impact. These results are consistent with our previous findings [31, 32]. However, no direct association was observed between these factors and the development of allergic diseases up to 24 months of age. We tested our hypotheses through subgroup analyses accounting for the direction and magnitude of the impact of these factors on the gut environment. A distinctive trend was identified between the early gut microbiota of Japanese infants and later development of allergic diseases, a relationship that has not been consistently observed in other studies.

Among infants who were not exposed to siblings or antibiotics, those who developed allergies had significantly lower gut microbiota diversity and *Bifidobacterium* abundance. Similar findings have been reported in studies conducted in Europe, the United States, Singapore, and Japan [43–47]. However, these studies did not consistently examine subgroup differences or consider the influence of early infancy factors. In contrast, in infants with siblings or AED, these external factors altered the gut microbiota during early infancy, and no significant differences in diversity or *Bifidobacterium* occupancy were observed between the allergic and non-allergic groups up to 24 months of age. Following weaning, the non-allergic group recovered its microbial diversity, whereas the allergic group maintained a low diversity and persistently high *Bifidobacterium* occupancy. Prior studies have also indicated a relationship between delayed gut microbiota maturation and the development of allergic diseases [48], although the research designs differed.

The primary objective of this study was to explore the relationship between the prevalence of dominant bacterial genera in Japanese infants and allergy development up to 24 months of age. Previous research has shown that *Bifidobacterium* are more prevalent in the gut microbiota of Japanese infants than in infants from other countries [14], a trend confirmed in our study, providing a rationale for examining the proportion and temporal changes in *Bifidobacterium*.

The comparison of *Bacteroides*, the second most prevalent genus, revealed that its occupancy differed after weaning. At 18 months of age, occupancy was lower in the allergy group than in the non-allergy group, whereas the opposite trend was observed in the subgroup without siblings at 24 months of age. However, there was no consistent longitudinal trend in *Bacteroides*, unlike *Bifidobacterium*, in relation to the onset of allergy. The logistic regression analysis revealed a significant association between *Bacteroides* occupancy at 12 months of age and the development of allergies, particularly recurrent wheezing, at 24 months of age. Although similar findings have been reported [30, 49], previous studies have not investigated subgroup differences, which may explain the inconsistencies across findings.

In addition to *Bifidobacterium* and *Bacteroides*, we examined butyrate-producing bacteria, including *Clostridium* and *Faecalibacterium*, as a secondary objective as they have been linked to allergy prevention [23, 43, 45, 47, 50]. Infants who developed allergies by 24 months of age had higher *Clostridium* occupancy, especially in those without siblings. Regarding *Faecalibacterium*, infants without allergies showed a higher occupancy rate after weaning, with significant differences observed at 24 months of age in the subgroup of infants with siblings who were not exposed to antibiotics. Previous reports have suggested associations between *Clostridium* and food allergies [36, 49, 51], and between *Faecalibacterium* and asthma [23, 45, 52], although the results are inconsistent. However, it is important to note that the *Clostridium* genus is taxonomically diverse; hence, there are limitations to interpreting results at the genus level. Additionally, as shown in the present study, *Faecalibacterium* is rarely observed to colonise the gastrointestinal tract in the early infant period, making it difficult to interpret its association with allergy onset at the same niche level as *Bifidobacterium*. Given these considerations, while these bacterial genera were analysed as secondary evaluation items, the results of the present study may be considered meaningful and useful for future evaluations at the species level, and for assessing the behaviour of these bacteria in the gut microbiota of early infancy.

Furthermore, in terms of the temporal changes in the relative abundance of *Clostridium*, at 9 months of age, the relative abundance in the allergic group appeared to be higher than that in the non-allergic group in both the overall and subgroup populations. However, at these time points, no significant differences in relative abundance were observed between the two groups. This trend is intriguing; however, no reasonable explanation for the difference in relative abundance at this age alone could be identified when compared with previous reports.

This study uniquely tracked the sequential trends of *Bacteroides*, *Clostridium*, and *Faecalibacterium*, focusing on the weaning period, and examined how the prevalence of these bacteria is linked to allergy development. The study’s comprehensive design, in which samples were regularly collected over 24 months, allowed for a thorough longitudinal analysis.

In summary, this study evaluated gut microbial diversity, with a primary focus on *Bifidobacterium* and *Bacteroides*, the dominant genera in Japanese infants up to 24 months of age. *Clostridium* and *Faecalibacterium*, the key butyrate-producing bacteria linked to allergies, were secondary targets. Our results suggest that maintaining normal *Bifidobacterium* proportions and microbial diversity in early infancy is crucial for reducing the risk of allergies. Additionally, our findings indicate that supporting the recovery of gut microbiota after disruptions may help mitigate allergy development.

The inconsistency in results across previous studies examining gut microbiota and allergies may be partly due to racial and regional differences [11–14]. However, it is possible that in studies focused on pre-weaning gut environments, the effects of external factors highlighted in this study may not have been fully considered.

In this study, careful inclusion and exclusion criteria were applied to ensure comparability of the infants’ gut environments. In the total analysis group, 43% of patients were diagnosed with allergic diseases, 33.9% with atopic dermatitis, 17.4% with recurrent wheezing, and 9.9% with food allergies. These rates are generally consistent with existing epidemiological data [53, 54], although the prevalence was slightly higher [55]. Given that 43% of the mothers had a history of allergies, this may have influenced the higher rates of atopic dermatitis. Nevertheless, the study design allowed for meaningful comparisons between the allergy and non-allergy groups, providing valuable insights into the underlying mechanisms of allergic disease development, while also accounting for maternal allergy history.

Subgroup and phenotype analyses revealed no significant differences in background factors between the allergy and non-allergy groups, ensuring valid comparisons. A unique aspect of this study was the exclusion of infants exposed to antibiotics before weaning, which limited the effects on delivery [29, 44, 46, 52, 56]. This design minimised external influences on the gut microbiota and enabled a meaningful comparison of differences between groups up to 24 months of age. Furthermore, although the timing of sample collection in previous studies varied [23, 29, 46, 48, 57–62], our study collected samples regularly, allowing for a longitudinal analysis of changes in the gut microbiota and their association with allergic disease development.

This study had a few limitations. First, because this was a prospective observational cohort study, causal relationships could not be established. Additionally, we did not measure metabolites, such as short-chain fatty acids, which are linked to allergy development, nor did we conduct functional evaluations of the intestinal environment. However, our findings may lay the groundwork for future studies with more controlled conditions, such as intervention trials that eliminate the effects of antimicrobial agents during early infancy. Second, this study was conducted in a single region of Japan, which limits the generalisability of the results. Further studies across different regions and countries are required to validate our findings. Third, our study was limited by its small sample size. Although the primary objective was to compare the presence or absence of allergic diseases for up to 24 months of age, a phenotypic analysis was conducted as a secondary objective. In infants of this age, allergic phenotypes are not independent, as they often overlap within the framework of the atopic march [63], and multiple phenotypes are included in the diagnostic criteria of each phenotype [39–42]. Additionally, some phenotypes, such as food allergies, are difficult to definitively diagnose up to 24 months of age [55]. In this study, 36.5% of the allergy cases exhibited two or more phenotypes, and similarities were observed in background factors and *Bifidobacterium* occupancy ratios across phenotypes. However, the small number of food allergy cases limited our ability to conduct robust subgroup analyses. A larger sample size is necessary to perform a comprehensive analysis of the various phenotypes.

Additionally, as the study was limited to external factors in the gut environment up to weaning, it may not have fully captured the diversity of the post-weaning intestinal environment. For example, although we evaluated butyrate-producing bacteria, such as *Clostridium* and *Faecalibacterium*, it is unclear how diet after weaning or oral drugs may have influenced these bacteria. Future research should include larger sample sizes to evaluate the post-weaning period and account for additional external factors, such as diet and medication use.

These limitations do not diminish the significance of the study’s findings but highlight the need for further research. Specifically, larger multicentre studies to investigate the relationship between changes in gut microbiota and the development of allergic diseases, and intervention trials that could provide more definitive insights, are warranted.

## Conclusions

Based on hypotheses defined in advance during the research planning stage and referencing the results of a pilot study, we investigated how exposure to antibiotics and the presence of older siblings influence long-term changes in major gut bacterial genera and allergy outcomes up to 24 months of age in a model that restricted external factors from infancy to weaning. In unstratified analyses, no overall differences were observed between infants with and without allergies in terms of *Bifidobacterium* abundance, alpha diversity, or beta diversity. However, when these two exposures were excluded and stratified, significant reductions in *Bifidobacterium* abundance and notable changes in beta diversity were observed in some subgroups of infants who developed allergies during the pre-weaning period. After the introduction of solid foods, microbial diversity recovered in both groups; however, recovery was significantly delayed in infants who developed allergies. These results suggest that the initial dominance of *Bifidobacterium* and the resilience of intestinal microbial diversity during the weaning transition period are important determinants of allergy risk. Clinically, the findings of this study suggest the importance of longitudinal monitoring of infant gut microbiota, considering perinatal antibiotic exposure and sibling history, and the development of personalized intervention strategies to maintain microbial diversity and reduce the incidence of allergic diseases.

## Electronic supplementary material

Below is the link to the electronic supplementary material.

Supplementary Material 1

Supplementary Material 2

## Data Availability

The data generated during the current study are available in the Figshare repository (DOI: 10.6084/m9.figshare.27940251), and the sequencing data is deposited in the DNA Data Bank of Japan (DDBJ; accession number: DRR236669-DRR237051, DRR640632-DRR641096).

## Abbreviations

AED: Antimicrobial exposure at delivery
CI: Confidence interval
mAPI: Modified Asthma Predictive Index
OR: Odds ratios
