# Supplementary material for: Impact of antimicrobial exposure at delivery and siblings on early Bifidobacterium succession and allergy development up to 24 months of age
Source: BMC Microbiol. 2025 May 28;25:332. doi: 10.1186/s12866-025-04056-7 (PMC12117752; doi:10.1186/s12866-025-04056-7)
Supplement: Supplementary file 1 — Supplementary Material 1 [file 12866_2025_4056_MOESM1_ESM.docx]

This survey is designed to examine the bacterial flora of Japanese infants and young children, and to help prevent infant diseases.

We would appreciate your cooperation.

- Please tell us about your child
- This questionnaire is for parents who have a lot of contact with their children on a regular basis.
- You can also answer this questionnaire on a computer or smartphone. Please access the questionnaire site using the URL or QR code below.

(The questionnaire format may differ slightly from this paper, but the content of the questions will be the same.)

| Date **of** entry: **20 /month**  Fill in person (please circle): mother father other guardian (relationship: ) | | | | | |
| --- | --- | --- | --- | --- | --- |
| Your child's  Date of birth | Please fill in the western calendar year | age | Full year Months | gender | Male / Female |
|  |  |  |  |  |  |

- Please answer the following items and the questionnaire items on the following pages and beyond.

Please circle (○) the number of the item that applies. Please fill in the fields provided, if any.

**<Click here if you want to answer on the web.**

URL address of this questionnaire.

https://questant.jp/q/Q9CCTCCT

QR code for this questionnaire.

　　　　　
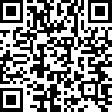


**Above is for dummies.**

Please answer about your child

**1. please answer the following questions about your child's health**

| **question** | **reply** | |
| --- | --- | --- |
| Has your doctor ever diagnosed your child as having asthma or possibly asthma? | 1. no. | 2. yes.  When did it start?  Do your child have a history of inhalation ?  (yes no) |
| Have your child ever been diagnosed by a doctor as atopic or possibly atopic? | 1. no. | 2. yes.  When did it start?  ...what medications have your child used?  ( ) |
| Has your doctor ever diagnosed your child with allergic conjunctivitis or possibly allergic conjunctivitis? | 1. no. | 2. yes.  When did it start?  ...what medications have your child used?  ( ) |
| Has your doctor ever diagnosed your child with allergic rhinitis or possibly allergic rhinitis? | 1. no. | 2. yes.  When did it start?  ...what medications have your child used?  ( ) |
| Does anyone in the family have allergies (including asthma, atopy, rhinitis, hay fever)? | 1. no. | 2. yes.  Who have it ?  ( )  ... What are the symptoms?  ( ) |
| Have your child ever had wheezing (wheezing, hissing, laboured breathing) unrelated to a cold or other infection? | 1. no. | 2. yes.  When did your child last have these symptoms?  ( )months old |
| Have your child had more than three wheezes in a year?  (Even wheezing over several days is counted as one event if it occurs continuously). | 1. no. | 2. yes.  How many times has this happened to your child ?  ( )times |
| Have your child been rubbing or scratching your skin constantly for the past year or so? | 1. no. | 2. yes.  When did it start?  How many times has this happened to your child ?  ( )times |
| Have your child been rubbing or scratching the skin constantly for the past week or so? | 1. no. | 2. yes. |
| Do your child rub or scratch the hollows of the elbows, knees, around the ankles, neck, eyes or cheeks? | 1. no. | 2. yes. |
| Overall over the past year, is your child's skin generally dry (flaky)? | 1. no. | 2. yes. |
| Do your child currently have eczema (reddened skin with dandruff, scabs, blisters or scratches on the surface) anywhere on the hollows of the elbows, knees, ankles, neck, eyes or cheeks? | 1. no. | 2. yes.  When did it start?  ( )months old |
| Have your child ever experienced allergic symptoms (e.g. urticaria, facial swelling, oral discomfort, paroxysmal cough, diarrhoea vomiting) after ingesting any food? | 1. no. | 2. yes.  Foods ingested ( )  When did it start?  Did you see a doctor at that time ?  (1. No 2. Yes)  ▪ If yes, have your child been diagnosed with a food allergy? (1. No 2. Yes) |
| Have your child ever had an allergy test (blood test)? | 1. no. | 2. yes.  Circle all allergens (positive by specific IgE determination or class 2 or above) identified by the test  Wheat Eggs Milk Milk Buckwheat Peanuts Shrimp Shrimp Crab Pollen House dust Dogs Cats Others ( ) |
| Ask if your child have never had an allergy test (blood test) before.  Would you be willing to have your child tested for allergies in the future if necessary? | 1. no. | 2. yes.  When would you like to be tested? Please circle the items that apply to you  I want to be tested immediately When the child grows up.  If symptoms occur At the start of nursery/preschool  If your doctor says you should take it.  Other ( ) |

That's all for the questionnaire. Please check again to make sure you have filled in all the information.

Thank you for your cooperation.
