# Supplementary material for: Impact of antimicrobial exposure at delivery and siblings on early Bifidobacterium succession and allergy development up to 24 months of age
Source: BMC Microbiol. 2025 May 28;25:332. doi: 10.1186/s12866-025-04056-7 (PMC12117752; doi:10.1186/s12866-025-04056-7)
Supplement: Supplementary file 2 — Supplementary Material 2 [file 12866_2025_4056_MOESM2_ESM.docx]

Supplementary Table S1. Comparison of background factors between groups diagnosed with allergic disease by 24 months and those not diagnosed.

| Allergy vs Non-Allergy |  |  |  |
| --- | --- | --- | --- |
|  | Allergy | non-Allergy | P value |
| Number of infants | 52 | 69 |  |
| Number of girls | 28(54) | 42(61) | 0.19 |
| Gestational age at birth | 274.8(8.1) | 275.8 (9.6) | 0.87 |
| Birth weight | 3046.7(357.6) | 3057.3 (318.5) | 0.05 |
| Antimicrobial exposure at delivery | 31(60) | 35(51) | 0.20 |
| Caesarean section | 11(21) | 14(20) | 0.07 |
| Infants with older siblings | 30(58) | 32(46) | 0.46 |
| Exclusive Breast feeding | 32(62) | 45(65) | 0.09 |
| Age of Mothers | 31.3(5.5) | 31.9(4.6) | 0.72 |
| Antimicrobial exposure after birth from 6mo | 30(58) | 34(49) | 0.46 |
| Antimicrobial exposure after birth from 6mo to 12mo | 15(29) | 20(29) | 1.00 |

For each factor, the number of each category variable and the percentage it represents of the total are displayed as percentages, and for continuous variables, the mean and standard deviation are indicated. To compare the presence or absence of allergy development in each factor, the Mann-Whitney U test was used for continuous variables, and Pearson's chi-square test was used for categorical variables.

Supplementary Table S2 . Comparison of background factors between groups diagnosed with allergic disease at 24 months of age and those not diagnosed with allergic disease by phenotypes of allergic disease.

|  | AED Subgroup | | | Non-AED Subgroup | | | Sib Subgroup | | | Non-Sib Subgroup | | | VD Subgroup | | |
| --- | --- | --- | --- | --- | --- | --- | --- | --- | --- | --- | --- | --- | --- | --- | --- |
|  | Allergy | non-Allergy | P | Allergy | non-Allergy | P | Allergy | non-Allergy | P | Allergy | non-Allergy | P | Allergy | non-Allergy | P |
| Number of infants | 31 | 35 |  | 21 | 34 |  | 30 | 32 |  | 22 | 37 |  | 41 | 55 |  |
| Number of girls | 14(45) | 21(60) | 0.32 | 14(67) | 21(62) | 0.78 | 16(53) | 18(56) | 1 | 12(55) | 24(65) | 0.58 | 21(51) | 34(61) | 0.4 |
| Gestational age at birth | 272.2  (8.7) | 272.5  (10.4) | 0.99 | 278.7  (5.0) | 279.2  (7.2) | 0.75 | 273.2  (9.1) | 272.4  (10.4) | 0.63 | 277.0  (5.9) | 278.8  (7.7) | 0.32 | 275.6  (5.7) | 275.6  (7.1) | 0.27 |
| Birth weight | 2973.2  (401.9) | 2998.5  (315.0) | 0.41 | 3155.1  (241.9) | 3117.9  (310.5) | 0.37 | 3032.8  (437.7) | 3055.6  (381.6) | 0.55 | 3065.5  (198.1) | 3058.8  (251.3) | 0.71 | 3095.9  (322.7) | 3095.9  (279.4) | 0.6 |
| AED | NA | NA | NA | NA | NA | NA | 20(67) | 20(63) | 0.8 | 11(50) | 15(41) | 0.50 | 20(49) | 21(38) | 0.4 |
| Caesarean section | 11(35) | 14(40) | 0.8 | NA | NA | NA | 9(30) | 11(34) | 0.79 | 2(9) | 3(8) | 1 | NA | NA | NA |
| Infants with older siblings | 20(65) | 20(57) | 0.62 | 10(48) | 12(35) | 0.41 | NA | NA | NA | NA | NA | NA | 21(51) | 21(38) | 0.22 |
| Exclusive Breast feeding | 18(58) | 19(54) | 0.81 | 14(67) | 26(76) | 0.54 | 20(67) | 21(66) | 1 | 12(55) | 24(65) | 0.58 | 25(61) | 38(69) | 0.52 |
| Age of Mothers | 31.9(5.1) | 32.8(4.2) | 0.43 | 30.3(5.8) | 31.0(4.7) | 0.79 | 32.7(4.9) | 33.1(4.6) | 0.84 | 29.4(5.5) | 30.9(4.2) | 0.21 | 30.9(5.5) | 30.9(4.5) | 0.58 |
| Antimicrobial exposure after birth from 6mo | 17(55) | 18(51) | 0.81 | 13(62) | 16(47) | 0.41 | 18(60) | 15(47) | 0.32 | 12(55) | 19(51) | 1 | 25(61) | 24(44) | 0.1 |
| Antimicrobial exposure after birth from 6mo to 12mo | 7(23) | 8(23) | 1.00 | 8(38) | 12(35) | 1.00 | 6(20) | 10(31) | 0.39 | 9(41) | 10(27) | 0.39 | 14(34) | 17(31) | 0.83 |

For each factor, the number of each category variable and the percentage it represents of the total are displayed as percentages. For continuous variables, the mean and standard deviation are indicated. To compare the presence or absence of allergy development in each factor, the Mann-Whitney U test was used for continuous variables, and Pearson‘s chi-square test was used for categorical variables. AED means infants group with antimicrobial exposure at delivery.

Supplementary Table S3 . Comparison of background factors between groups diagnosed with allergic disease at 24 months of age and those not diagnosed with allergic disease by phenotypes of allergic disease.

|  | Atopic Dermatitis | non-Allergy | P value |  | Reccurent Wheezing | non-Allergy | P value |  | Food Allergy | non-Allergy | P value |
| --- | --- | --- | --- | --- | --- | --- | --- | --- | --- | --- | --- |
| Number of infants | 41 | 69 |  |  | 21 | 69 |  |  | 12 | 69 |  |
| Number of girls | 23(56) | 42(61) | 0.69 |  | 11(52) | 42(61) | 0.61 |  | 5(42) | 42(61) | 0.34 |
| Gestational age at birth | 274.8(8.1) | 275.8 (9.6) | 0.48 |  | 274.4(7.7) | 275.8 (9.6) | 0.51 |  | 274.0  (7.2) | 275.8 (9.6) | 0.52 |
| Birth weight | 3048.8(337.5) | 3057.3 (318.5) | 0.70 |  | 3054.7  (440.5) | 3057.3 (318.5) | 0.71 |  | 3044.7  (236.0) | 3057.3 (318.5) | 0.88 |
| AED | 26(63) | 35(51) | 0.24 |  | 14(67) | 35(51) | 0.22 |  | 6(50) | 35(51) | 1.00 |
| Caesarean section | 9(22) | 14(20) | 1.00 |  | 4(19) | 14(20) | 1.00 |  | 2(17) | 14(20) | 1.00 |
| Infants with older siblings | 23(56) | 32(46) | 0.43 |  | 14(67) | 32(46) | 0.14 |  | 9(75) | 32(46) | 0.16 |
| Exclusive Breast feeding | 24(59) | 45(65) | 0.54 |  | 12(57) | 45(65) | 0.61 |  | 9(75) | 45(65) | 0.74 |
| Age of Mothers | 30.7(5.4) | 31.9(4.6) | 0.25 |  | 31.7(4.9) | 31.9(4.6) | 0.73 |  | 30.0  (6.2) | 31.9  (4.6) | 0.35 |
| Antimicrobial exposure after birth from 6mo | 21(51) | 34(49) | 1.00 |  | 14(67) | 34(49) | 0.21 |  | 10(83) | 34(49) | 0.06 |
| Antimicrobial exposure after birth from 6mo to 12mo | 11(27) | 20(29) | 1.00 |  | 8(38) | 20(29) | 0.43 |  | 4(33) | 20(29) | 0.74 |

For each factor, the number of each category variable and the percentage it represents of the total are displayed as percentages. For continuous variables, the mean and standard deviation are indicated. To compare the presence or absence of allergy development in each factor, the Mann-Whitney U test was used for continuous variables, and Pearson‘s chi-square test was used for categorical variables. AED means infants group with antimicrobial exposure at delivery.

Supplementary Table S4 . The influence of each background factor on the development of allergies up to 24 months of age by subgroups.

|  | **AED Subgroup (N=66)** | | |  | **Non-AED Subgroup (N=55)** | | |  | **Sib Subgroup (N=62)** | | |  | **Non-Sib Subgroup(N=59)** | | |  | **VD Subgroup (N=96)** | | |  |
| --- | --- | --- | --- | --- | --- | --- | --- | --- | --- | --- | --- | --- | --- | --- | --- | --- | --- | --- | --- | --- |
|  | Odds Ratios | 95%CI |  |  | Odds Ratios | 95%CI |  |  | Odds Ratios | 95%CI |  |  | Odds Ratios | 95%CI |  |  | Odds Ratios | 95%CI |  |  |
| Gestational days | 1.01 | 0.93 | 1.1 |  | 0.974 | 0.882 | 1.075 |  | 1.024 | 0.923 | 1.136 |  | 0.966 | 0.883 | 1.056 |  | 0.947 | 0.877 | 1.022 |  |
| Birth weight | 1 | 0.999 | 1.002 |  | 1.001 | 0.999 | 1.003 |  | 1 | 0.998 | 1.002 |  | 1 | 0.997 | 1.003 |  | 1.001 | 0.999 | 1.003 |  |
| Age of mothers | 1.05 | 0.94 | 1.18 |  | 0.954 | 0.845 | 1.077 |  | 0.979 | 0.875 | 1.096 |  | 0.912 | 0.807 | 1.03 |  | 0.951 | 0.866 | 1.044 |  |
| Feale | 2.1 | 0.72 | 6.16 |  | 1.533 | 0.438 | 5.366 |  | 1.072 | 0.34 | 3.384 |  | 0.521 | 0.144 | 1.876 |  | 0.704 | 0.279 | 1.774 |  |
| Vaginal delivery | 0.53 | 0.11 | 2.46 |  | NA | NA | NA |  | 0.546 | 0.075 | 3.991 |  | 0.599 | 0.056 | 6.449 |  | 0.536 | 0.209 | 1.373 |  |
| Infants without siblings | 1.4 | 0.42 | 4.65 |  | 0.599 | 0.172 | 2.083 |  | 1.473 | 0.379 | 5.723 |  | 0.774 | 0.218 | 2.743 |  | 0.813 | 0.325 | 2.038 |  |
| Mothers without allergy | 1.75 | 0.58 | 5.32 |  | 0.553 | 0.157 | 1.943 |  | 1.698 | 0.511 | 5.649 |  | 0.397 | 0.099 | 1.591 |  | 0.337 | 0.127 | 0.892 | * |
| Exclusively breast-fed | 0.92 | 0.31 | 2.77 |  | 0.573 | 0.155 | 2.118 |  | 1.079 | 0.336 | 3.467 |  | 0.504 | 0.139 | 1.835 |  | 0.569 | 0.216 | 1.494 |  |
| Without Antimicrobial exposure after 6m.o. | 1.37 | 0.41 | 4.6 |  | 0.356 | 0.069 | 1.844 |  | 3.819 | 0.984 | 14.827 |  | 1.859 | 0.36 | 9.595 |  | 0.367 | 0.111 | 1.211 |  |
| Without Antimicrobial exposure after 6mo to 12mo | 0.77 | 0.18 | 3.29 |  | 1.926 | 0.369 | 10.046 |  | 0.226 | 0.048 | 1.053 |  | 0.322 | 0.053 | 1.968 |  | 1.952 | 0.547 | 6.962 |  |

The odds ratio and 95% confidence interval were calculated using logistic regression analysis. The significance level was set at 5%. *p < 0.05.　AED means infants group with antimicrobial exposure at delivery.

Supplementary Table S5. The influence of background factors on the degree of dominance of each bacterial genus in each month age.

|  | **Bifidobacterium** | | |  | **Bacteroides** | | |  | **Clostridium** | | |  | **Faecalibacterium** | | |  |
| --- | --- | --- | --- | --- | --- | --- | --- | --- | --- | --- | --- | --- | --- | --- | --- | --- |
| **1month** | Odds Ratios | 95%CI |  |  | Odds Ratios | 95%CI |  |  | Odds Ratios | 95%CI |  |  | Odds Ratios | 95%CI |  |  |
| Age of Mothers | 0.989 | 0.91 | 1.076 |  | 1.012 | 0.923 | 1.111 |  | 1.062 | 0.959 | 1.177 |  | NA |  |  |  |
| Gestational days | 0.971 | 0.91 | 1.035 |  | 1.071 | 0.988 | 1.161 |  | 0.978 | 0.909 | 1.053 |  | NA |  |  |  |
| Birth weight | 1 | 1 | 1.002 |  | 1 | 0.999 | 1.002 |  | 1 | 0.998 | 1.001 |  | NA |  |  |  |
| Infants without Allergy at 24 month | 1.997 | 0.89 | 4.493 |  | 1.566 | 0.672 | 3.652 |  | 1.632 | 0.634 | 4.2 |  | NA |  |  |  |
| Female | 0.875 | 0.39 | 1.966 |  | 1.175 | 0.504 | 2.738 |  | 0.614 | 0.237 | 1.592 |  | NA |  |  |  |
| Vaginal Delivery | 0.553 | 0.18 | 1.681 |  | 13.106 | 3.185 | 53.936 | *** | 0.121 | 0.03 | 0.48 | ** | NA |  |  |  |
| Infants without siblings | 0.336 | 0.14 | 0.797 | * | 1.011 | 0.426 | 2.404 |  | 3.923 | 1.333 | 11.541 | * | NA |  |  |  |
| Non-AED | 2.89 | 1.14 | 7.349 | * | 1.487 | 0.6 | 3.685 |  | 0.233 | 0.08 | 0.675 | ** | NA |  |  |  |
| Mothers without allergy | 0.861 | 0.37 | 2.02 |  | 0.888 | 0.318 | 2.477 |  | 1.626 | 0.574 | 4.601 |  | NA |  |  |  |
| Exclusively breast-fed | 2.307 | 1 | 5.304 |  | 0.682 | 0.28 | 1.661 |  | 0.197 | 0.075 | 0.517 | ** | NA |  |  |  |
|  |  |  |  |  |  |  |  |  |  |  |  |  |  |  |  |  |
| **3month** | Odds Ratios | 95%CI |  |  | Odds Ratios | 95%CI |  |  | Odds Ratios | 95%CI |  |  | Odds Ratios | 95%CI |  |  |
| Age of Mothers | 0.986 | 0.91 | 1.07 |  | 1.004 | 0.921 | 1.094 |  | 1.004 | 0.914 | 1.103 |  |  |  |  |  |
| Gestational days | 1.002 | 0.94 | 1.068 |  | 1.032 | 0.966 | 1.104 |  | 1.004 | 0.936 | 1.077 |  |  |  |  |  |
| Birth weight | 1.001 | 1 | 1.002 |  | 1.001 | 0.999 | 1.002 |  | 0.999 | 0.998 | 1.001 |  |  |  |  |  |
| Infants without Allergy at 24 month | 2.386 | 1.07 | 5.329 | * | 1.575 | 0.686 | 3.617 |  | 0.864 | 0.362 | 2.065 |  | NA |  |  |  |
| Female | 0.729 | 0.33 | 1.626 |  | 1.123 | 0.489 | 2.581 |  | 0.854 | 0.353 | 2.07 |  | NA |  |  |  |
| Vaginal Delivery | 0.74 | 0.25 | 2.207 |  | 15.016 | 2.985 | 75.524 | *** | 0.57 | 0.173 | 1.875 |  | NA |  |  |  |
| Infants without siblings | 0.286 | 0.12 | 0.676 | ** | 1.357 | 0.59 | 3.122 |  | 2.051 | 0.786 | 5.352 |  | NA |  |  |  |
| Non-AED | 3.134 | 1.24 | 7.947 | * | 1.469 | 0.61 | 3.536 |  | 0.129 | 0.047 | 0.353 | *** | NA |  |  |  |
| Mothers without allergy | 0.849 | 0.37 | 1.951 |  | 1.007 | 0.414 | 2.45 |  | 2.036 | 0.774 | 5.351 |  |  |  |  |  |
| Exclusively breast-fed | 0.957 | 0.42 | 2.188 |  | 0.56 | 0.229 | 1.371 |  | 0.311 | 0.126 | 0.767 | * | NA |  |  |  |
|  |  |  |  |  |  |  |  |  |  |  |  |  |  |  |  |  |
| **6month** | Odds Ratios | 95%CI |  |  | Odds Ratios | 95%CI |  |  | Odds Ratios | 95%CI |  |  | Odds Ratios | 95%CI |  |  |
| Age of Mothers | 0.935 | 0.86 | 1.013 |  | 1.013 | 0.935 | 1.097 |  | 0.989 | 0.912 | 1.073 |  |  |  |  |  |
| Gestational days | 0.98 | 0.92 | 1.042 |  | 0.99 | 0.93 | 1.054 |  | 0.974 | 0.914 | 1.037 |  |  |  |  |  |
| Birth weight | 0.999 | 1 | 1.001 |  | 1.001 | 1 | 1.002 |  | 1 | 0.999 | 1.001 |  |  |  |  |  |
| Infants without Allergy at 24 month | 1.381 | 0.65 | 2.916 |  | 0.821 | 0.383 | 1.757 |  | 0.56 | 0.254 | 1.237 |  | NA |  |  |  |
| Female | 0.497 | 0.23 | 1.061 |  | 2.041 | 0.942 | 4.426 |  | 0.531 | 0.244 | 1.157 |  | NA |  |  |  |
| Vaginal Delivery | 1.185 | 0.42 | 3.385 |  | 2.418 | 0.815 | 7.174 |  | 1.684 | 0.755 | 3.753 |  | NA |  |  |  |
| Infants without siblings | 0.724 | 0.33 | 1.577 |  | 0.592 | 0.267 | 1.316 |  | 1.017 | 0.345 | 3.004 |  | NA |  |  |  |
| Non-AED | 1.168 | 0.5 | 2.738 |  | 1.202 | 0.508 | 2.848 |  | 0.602 | 0.266 | 1.358 |  | NA |  |  |  |
| Mothers without allergy | 1.358 | 0.6 | 3.056 |  | 0.687 | 0.302 | 1.567 |  | 0.521 | 0.215 | 1.259 |  |  |  |  |  |
| Exclusively breast-fed | 1.315 | 0.6 | 2.869 |  | 0.489 | 0.218 | 1.094 |  | 0.357 | 0.157 | 0.813 | * | NA |  |  |  |
|  |  |  |  |  |  |  |  |  |  |  |  |  |  |  |  |  |
| **9month** | Odds Ratios | 95%CI |  |  | Odds Ratios | 95%CI |  |  | Odds Ratios | 95%CI |  |  | Odds Ratios | 95%CI |  |  |
| Age of Mothers | 0.964 | 0.89 | 1.045 |  | 1.062 | 0.979 | 1.152 |  | 1.003 | 0.926 | 1.086 |  |  |  |  |  |
| Gestational days | 1.018 | 0.96 | 1.084 |  | 0.978 | 0.917 | 1.042 |  | 1 | 0.94 | 1.063 |  |  |  |  |  |
| Birth weight | 1 | 1 | 1.001 |  | 1 | 0.999 | 1.001 |  | 1 | 0.999 | 1.001 |  |  |  |  |  |
| Infants without Allergy at 24 month | 1.278 | 0.59 | 2.777 |  | 0.993 | 0.461 | 2.138 |  | 0.618 | 0.286 | 1.333 |  | NA |  |  |  |
| Female | 0.731 | 0.33 | 1.609 |  | 2.118 | 0.972 | 4.615 |  | 0.661 | 0.304 | 1.437 |  | NA |  |  |  |
| Vaginal Delivery | 0.604 | 0.2 | 1.796 |  | 4.733 | 1.515 | 14.79 | ** | 0.238 | 0.076 | 0.747 | * | NA |  |  |  |
| Infants without siblings | 1.013 | 0.45 | 2.278 |  | 0.904 | 0.407 | 2.009 |  | 1.049 | 0.47 | 2.338 |  | NA |  |  |  |
| Non-AED | 1.52 | 0.63 | 3.676 |  | 0.611 | 0.258 | 1.45 |  | 1.387 | 0.584 | 3.295 |  | NA |  |  |  |
| Mothers without allergy | 1.089 | 0.47 | 2.508 |  | 0.672 | 0.293 | 1.543 |  | 1.188 | 0.522 | 2.702 |  |  |  |  |  |
| Exclusively breast-fed | 3.933 | 1.72 | 8.977 | * | 1.062 | 0.478 | 2.359 |  | 0.559 | 0.251 | 1.243 |  | NA |  |  |  |
|  |  |  |  |  |  |  |  |  |  |  |  |  |  |  |  |  |
| **12month** | Odds Ratios | 95%CI |  |  | Odds Ratios | 95%CI |  |  | Odds Ratios | 95%CI |  |  | Odds Ratios | 95%CI |  |  |
| Age of Mothers | 1.012 | 0.93 | 1.098 |  | 1.022 | 0.936 | 1.116 |  | 0.989 | 0.91 | 1.074 |  | 0.959 | 0.876 | 1.051 |  |
| Gestational days | 1.022 | 0.96 | 1.09 |  | 0.993 | 0.928 | 1.063 |  | 1.015 | 0.951 | 1.083 |  | 0.994 | 0.927 | 1.066 |  |
| Birth weight | 1 | 1 | 1.001 |  | 0.999 | 0.998 | 1.001 |  | 1.001 | 0.999 | 1.002 |  | 0.999 | 0.997 | 1 |  |
| Infants without Allergy at 24 month | 0.421 | 0.19 | 0.932 | * | 2.718 | 1.162 | 6.354 | * | 0.833 | 0.38 | 1.826 |  | 1.015 | 0.44 | 2.34 |  |
| Female | 0.787 | 0.36 | 1.743 |  | 2.607 | 1.11 | 6.126 | * | 0.71 | 0.324 | 1.557 |  | 0.725 | 0.313 | 1.679 |  |
| Vaginal Delivery | 0.647 | 0.21 | 1.976 |  | 4.349 | 1.293 | 14.623 | * | 0.537 | 0.18 | 1.609 |  | 0.684 | 0.218 | 2.142 |  |
| Infants without siblings | 0.819 | 0.36 | 1.852 |  | 1.317 | 0.56 | 3.097 |  | 0.966 | 0.425 | 2.195 |  | 0.601 | 0.248 | 1.454 |  |
| Non-AED | 0.677 | 0.27 | 1.69 |  | 0.526 | 0.2 | 1.387 |  | 2.338 | 0.933 | 5.856 |  | 0.951 | 0.357 | 2.534 |  |
| Mothers without allergy | 0.836 | 0.37 | 1.914 |  | 2.594 | 1.038 | 6.482 |  | 0.431 | 0.18 | 1.028 |  | 2.772 | 1.021 | 7.529 | * |
| Exclusively breast-fed | 2.745 | 1.18 | 6.377 | * | 0.275 | 0.111 | 0.681 | ** | 0.364 | 0.157 | 0.841 | * | 0.919 | 0.384 | 2.196 |  |
| Without Abx exposure after 6mo to 12mo | 0.718 | 0.31 | 1.69 |  | 0.627 | 0.258 | 1.522 |  | 0.524 | 0.222 | 1.24 |  | 0.429 | 0.176 | 1.042 |  |
|  |  |  |  |  |  |  |  |  |  |  |  |  |  |  |  |  |
| **18month** | Odds Ratios | 95%CI |  |  | Odds Ratios | 95%CI |  |  | Odds Ratios | 95%CI |  |  | Odds Ratios | 95%CI |  |  |
| Age of Mothers | 0.996 | 0.92 | 1.079 |  | 0.955 | 0.878 | 1.038 |  | 1 | 0.6485 | 0.4207 |  | 0.936 | 0.863 | 1.016 |  |
| Gestational days | 1.038 | 0.98 | 1.104 |  | 0.996 | 0.935 | 1.061 |  | 1 | 0.3915 | 0.5315 |  | 0.967 | 0.907 | 1.03 |  |
| Birth weight | 1 | 1 | 1.002 |  | 0.999 | 0.998 | 1.001 |  | 1 | 0.0005 | 0.983 |  | 1 | 0.999 | 1.001 |  |
| Infants without Allergy at 24 month | 0.554 | 0.25 | 1.209 |  | 1.89 | 0.858 | 4.165 |  | 0.727 | 0.339 | 1.559 |  | 1.269 | 0.58 | 2.779 |  |
| Female | 1.524 | 0.69 | 3.351 |  | 0.563 | 0.252 | 1.254 |  | 1.193 | 0.552 | 2.578 |  | 0.567 | 0.258 | 1.246 |  |
| Vaginal Delivery | 1.566 | 0.52 | 4.706 |  | 0.735 | 0.242 | 2.228 |  | 1.776 | 0.612 | 5.149 |  | 0.856 | 0.287 | 2.551 |  |
| Infants without siblings | 0.5 | 0.22 | 1.132 |  | 2.349 | 1.038 | 5.318 | * | 0.908 | 0.41 | 2.011 |  | 0.486 | 0.217 | 1.092 |  |
| Non-AED | 1.251 | 0.51 | 3.048 |  | 1.648 | 0.666 | 4.076 |  | 0.528 | 0.219 | 1.271 |  | 0.711 | 0.291 | 1.737 |  |
| Mothers without allergy | 1.037 | 0.45 | 2.369 |  | 1.39 | 0.602 | 3.21 |  | 1 | 0.4218 | 0.516 |  | 1.541 | 0.665 | 3.573 |  |
| Exclusively breast-fed | 1.624 | 0.72 | 3.648 |  | 0.592 | 0.26 | 1.348 |  | 0.706 | 0.318 | 1.567 |  | 1.351 | 0.596 | 3.06 |  |
| Without Abx exposure after 6mo to 12mo | 0.763 | 0.33 | 1.788 |  | 1.028 | 0.432 | 2.446 |  | 0.595 | 0.255 | 1.388 |  | 0.474 | 0.198 | 1.135 |  |
|  |  |  |  |  |  |  |  |  |  |  |  |  |  |  |  |  |
| **24month** | Odds Ratios | 95%CI |  |  | Odds Ratios | 95%CI |  |  | Odds Ratios | 95%CI |  |  | Odds Ratios | 95%CI |  |  |
| Age of Mothers | 0.956 | 0.87 | 1.044 |  | 0.993 | 0.915 | 1.078 |  | 1.009 | 0.93 | 1.095 |  | 0.975 | 0.898 | 1.06 |  |
| Gestational days | 1.015 | 0.95 | 1.082 |  | 0.98 | 0.921 | 1.043 |  | 0.938 | 0.875 | 1.006 |  | 0.917 | 0.854 | 0.984 |  |
| Birth weight | 1.002 | 1 | 1.003 |  | 0.999 | 0.998 | 1.001 |  | 1.001 | 1 | 1.003 |  | 1 | 0.998 | 1.001 |  |
| Infants without Allergy at 24 month | 0.45 | 0.2 | 1.014 |  | 0.8 | 0.363 | 1.766 |  | 1.491 | 0.674 | 3.299 |  | 1.344 | 0.616 | 2.931 |  |
| Female | 1.74 | 0.78 | 3.877 |  | 0.747 | 0.341 | 1.636 |  | 0.628 | 0.286 | 1.378 |  | 0.975 | 0.45 | 2.115 |  |
| Vaginal Delivery | 1.618 | 0.52 | 5.031 |  | 1.272 | 0.417 | 3.881 |  | 2.282 | 0.739 | 7.05 |  | 1.149 | 0.382 | 3.455 |  |
| Infants without siblings | 0.507 | 0.22 | 1.157 |  | 1.209 | 0.538 | 2.719 |  | 0.584 | 0.26 | 1.308 |  | 1.103 | 0.499 | 2.436 |  |
| Non-AED | 1.401 | 0.56 | 3.518 |  | 0.488 | 0.199 | 1.2 |  | 0.527 | 0.214 | 1.298 |  | 0.703 | 0.289 | 1.711 |  |
| Mothers without allergy | 0.399 | 0.16 | 0.992 |  | 1.257 | 0.534 | 2.962 |  | 1.291 | 0.547 | 3.048 |  | 1.789 | 0.743 | 4.307 |  |
| Exclusively breast-fed | 1.038 | 0.45 | 2.404 |  | 0.526 | 0.23 | 1.205 |  | 1.246 | 0.54 | 2.874 |  | 1.175 | 0.519 | 2.66 |  |
| Without Abx exposure after 6m.o. | 1.151 | 0.42 | 3.147 |  | 0.901 | 0.335 | 2.422 |  | 1.3 | 0.485 | 3.483 |  | 0.948 | 0.36 | 2.497 |  |
| Without Abx exposure after 6mo to 12mo | 1.729 | 0.57 | 5.241 |  | 0.777 | 0.263 | 2.293 |  | 0.957 | 0.326 | 2.811 |  | 0.488 | 0.167 | 1.424 |  |

To investigate the relationship between each background factor and the occupancy of each bacterial species, the occupancy of each bacterial species was divided into two groups by the median, and the odds ratio and 95% confidence interval were calculated by logistic regression analysis. The significance level was set at 5%.. *p < 0.05, **p < 0.01, ***p < 0.001.　AED means infants group with antimicrobial exposure at delivery. NA represents those for which occupancy was not analyzed for comparison due to the large number of individuals with zero occupancy in either group.

Supplementary Table S6 . Comparison of diversity by presence or absence of allergy development in all age groups (all analysis target populations and each subgroup).

| All analysis target population | Non Allergy vs Allergy | 1m.o. | 3 m.o. | 6 m.o. | 9 m.o. | 12 m.o. | 18 m.o. | 24 m.o. |
| --- | --- | --- | --- | --- | --- | --- | --- | --- |
| αdiversity | Chao1 | 0.42 | 0.77 | 0.19 | 0.64 | 0.22 | 0.75 | 0.68 |
|  | Shannon | 0.44 | 0.86 | 0.13 | 0.16 | 0.054 | 0.71 | 0.1 |
| βdiversity | Unweighted | 0.29 | 0.6 | 0.44 | 0.08 | 0.94 | 0.54 | 0.25 |
|  | Weighted | 0.28 | 0.46 | 0.41 | 0.07 | 0.17 | 0.15 | 0.24 |
|  |  |  |  |  |  |  |  |  |
| AED subgroup | Non Allergy vs Allergy | 1m.o. | 3 m.o. | 6 m.o. | 9 m.o. | 12 m.o. | 18 m.o. | 24 m.o. |
| αdiversity | Chao1 | 0.61 | 0.37 | 0.091 | 0.13 | 0.045 | 0.63 | 0.43 |
|  | Shannon | 0.94 | 0.98 | 0.61 | 0.1 | 0.038 | 0.64 | 0.042 |
| βdiversity | Unweighted | 0.93 | 0.97 | 0.59 | 0.55 | 0.57 | 0.41 | 0.38 |
|  | Weighted | 0.87 | 0.88 | 0.29 | 0.16 | 0.03 | 0.72 | 0.15 |
|  |  |  |  |  |  |  |  |  |
| Non-AED subgroup | Non Allergy vs Allergy | 1m.o. | 3 m.o. | 6 m.o. | 9 m.o. | 12 m.o. | 18 m.o. | 24 m.o. |
| αdiversity | Chao1 | 0.42 | 0.46 | 0.68 | 0.47 | 0.76 | 0.79 | 0.74 |
|  | Shannon | 0.33 | 0.74 | 0.08 | 0.7 | 0.53 | 0.82 | 0.28 |
| βdiversity | Unweighted | 0.58 | 0.14 | 0.26 | 0.19 | 0.99 | 0.98 | 0.54 |
|  | Weighted | 0.2 | 0.23 | 0.82 | 0.09 | 0.96 | 0.16 | 0.49 |
|  |  |  |  |  |  |  |  |  |
| Sib subgroup | Non Allergy vs Allergy | 1m.o. | 3 m.o. | 6 m.o. | 9 m.o. | 12 m.o. | 18 m.o. | 24 m.o. |
| αdiversity | Chao1 | 0.39 | 0.91 | 0.41 | 0.94 | 0.7 | 0.71 | 0.37 |
|  | Shannon | 0.68 | 0.82 | 0.99 | 0.089 | 0.18 | 0.15 | 0.013 |
| βdiversity | Unweighted | 0.78 | 0.99 | 0.48 | 0.4 | 0.8 | 0.15 | 0.17 |
|  | Weighted | 0.78 | 0.61 | 0.31 | 0.1 | 0.15 | 0.27 | 0.06 |
|  |  |  |  |  |  |  |  |  |
| Non-Sib subgroup | Non Allergy vs Allergy | 1m.o. | 3 m.o. | 6 m.o. | 9 m.o. | 12 m.o. | 18 m.o. | 24 m.o. |
| αdiversity | Chao1 | 0.77 | 0.83 | 0.28 | 0.4 | 0.2 | 0.42 | 0.62 |
|  | Shannon | 0.32 | 0.8 | 0.011 | 0.93 | 0.36 | 0.24 | 0.81 |
| βdiversity | Unweighted | 0.18 | 0.036 | 0.12 | 0.26 | 0.71 | 0.99 | 0.89 |
|  | Weighted | 0.009 | 0.025 | 0.65 | 0.007 | 0.83 | 0.88 | 0.45 |
|  |  |  |  |  |  |  |  |  |
| Non-AED + Sib subgroup | Non Allergy vs Allergy | 1m.o. | 3 m.o. | 6 m.o. | 9 m.o. | 12 m.o. | 18 m.o. | 24 m.o. |
| αdiversity | Chao1 | 0.21 | 0.34 | 0.77 | 0.72 | 0.62 | 0.21 | 0.26 |
|  | Shannon | 0.52 | 0.57 | 0.43 | 0.12 | 0.48 | 0.21 | 0.065 |
| βdiversity | Unweighted | 0.57 | 0.99 | 0.99 | 0.74 | 0.9 | 0.28 | 0.31 |
|  | Weighted | 0.68 | 0.51 | 0.72 | 0.39 | 0.48 | 0.09 | 0.006 |
|  |  |  |  |  |  |  |  |  |
| AED+ Non-Sib subgroup | Non Allergy vs Allergy | 1m.o. | 3 m.o. | 6 m.o. | 9 m.o. | 12 m.o. | 18 m.o. | 24 m.o. |
| αdiversity | Chao1 | 0.9 | 0.74 | 0.24 | 0.05 | 0.05 | 0.89 | 0.38 |
|  | Shannon | 0.62 | 0.59 | 0.1 | 0.22 | 0.08 | 0.82 | 0.38 |
| βdiversity | Unweighted | 0.28 | 0.051 | 0.38 | 0.26 | 0.096 | 0.96 | 0.87 |
|  | Weighted | 0.18 | 0.42 | 0.68 | 0.12 | 0.18 | 0.97 | 0.24 |
|  |  |  |  |  |  |  |  |  |
| Non-AED+ Non-Sib subgroup | Non Allergy vs Allergy | 1m.o. | 3 m.o. | 6 m.o. | 9 m.o. | 12 m.o. | 18 m.o. | 24 m.o. |
| αdiversity | Chao1 | 0.77 | 0.79 | 0.44 | 0.41 | 0.9 | 0.45 | 0.22 |
|  | Shannon | 0.37 | 0.94 | 0.0997 | 0.3 | 0.74 | 0.26 | 0.58 |
| βdiversity | Unweighted | 0.32 | 0.024 | 0.17 | 0.25 | 0.19 | 0.85 | 0.57 |
|  | Weighted | 0.014 | 0.02 | 0.22 | 0.06 | 0.63 | 0.68 | 0.58 |

The p-values for the results of the Shannon and Chao1 analyses for alpha diversity and the weighted and unweighted analyses for beta diversity are listed for each month of age for the entire analysis population and each subgroup, comparing the results for the allergy-onset group and non-onset group. AED and Non-AED indicate infants with and without antimicrobial exposure at delivery, respectively. Sib and Non-Sib indicate infants with and without older siblings, respectively.

Supplementary Table S7 . Influence of each background factor on the development of allergies up to 24 months of age by phenotypes.

|  | **Atopic Dermatitis (N=41)**  **vs Non-Allergy (N=69)** | | |  | **Recurrent Wheezing (N=21)**  **vs Non-Allergy (N=69)** | | |  |  | **Food Allergy (N=12)**  **vs Non-Allergy (N=69)** | | |  |
| --- | --- | --- | --- | --- | --- | --- | --- | --- | --- | --- | --- | --- | --- |
|  | Odds Ratios | 95%CI |  |  | Odds Ratios | 95%CI |  |  |  | Odds Ratios | 95%CI |  |  |
| Gestational days | 0.986 | 0.923 | 1.053 |  | 0.964 | 0.882 | 1.054 |  |  | 0.952 | 0.828 | 1.095 |  |
| Birth weight | 1 | 0.999 | 1.002 |  | 1 | 0.998 | 1.002 |  |  | 1 | 0.996 | 1.004 |  |
| Age of mothers | 0.931 | 0.853 | 1.017 |  | 0.933 | 0.824 | 1.056 |  |  | 0.825 | 0.691 | 0.985 |  |
| Feale | 0.963 | 0.409 | 2.264 |  | 0.69 | 0.226 | 2.106 |  |  | 0.391 | 0.08 | 1.895 |  |
| Vaginal delivery | 1.777 | 0.426 | 7.422 |  | 8.865 | 0.958 | 82.032 |  |  | 9.042 | 0.417 | 196.13 |  |
| Infants without siblings | 0.593 | 0.241 | 1.455 |  | 0.288 | 0.083 | 0.998 | * |  | 0.136 | 0.02 | 0.915 | * |
| Non-AED | 0.579 | 0.229 | 1.466 |  | 0.454 | 0.132 | 1.558 |  |  | 0.587 | 0.088 | 3.928 |  |
| Mothers without allergy | 0.566 | 0.235 | 1.364 |  | 0.438 | 0.132 | 1.455 |  |  | 0.346 | 0.059 | 2.016 |  |
| Exclusively breast-fed | 0.688 | 0.283 | 1.672 |  | 0.86 | 0.284 | 2.611 |  |  | 2.921 | 0.458 | 18.649 |  |
| Without Antimicrobial exposure after 6m.o. | 0.863 | 0.304 | 2.451 |  | 0.275 | 0.061 | 1.245 |  |  | 0.06 | 0.007 | 0.506 | * |
| Without Antimicrobial exposure after 6mo to 12mo | 1.18 | 0.367 | 3.799 |  | 1.509 | 0.344 | 6.615 |  |  | 2.616 | 0.394 | 17.375 |  |

The odds ratio and 95% confidence interval were calculated using logistic regression analysis. The significance level was set at 5%. *p < 0.05.　AED means infants group with antimicrobial exposure at delivery.

Supplementary Table S8 . Comparison of background factors between groups diagnosed with each phenotype of allergic disease by 24 months and those not diagnosed.

|  | **Atopic Dermatitis** | non-Allergy | P value |  | **Reccurent Wheezing** | non-Allergy | P value |  | **Food Allergy** | non-Allergy | P value |
| --- | --- | --- | --- | --- | --- | --- | --- | --- | --- | --- | --- |
| Number of infants | 41 | 69 |  |  | 21 | 69 |  |  | 12 | 69 |  |
| Number of girl | 23(56) | 42(61) | 0.69 |  | 11(52) | 42(61) | 0.61 |  | 5(42) | 42(61) | 0.34 |
| Gestational age at birth | 274.8(8.1) | 275.8 (9.6) | 0.48 |  | 274.4(7.7) | 275.8 (9.6) | 0.51 |  | 274.0(7.2) | 275.8 (9.6) | 0.52 |
| Birth weight | 3048.8(337.5) | 3057.3 (318.5) | 0.70 |  | 3054.7(440.5) | 3057.3 (318.5) | 0.71 |  | 3044.7(236.0) | 3057.3 (318.5) | 0.88 |
| Antimicrobial exposure at delivery | 26(63) | 35(51) | 0.24 |  | 14(67) | 35(51) | 0.22 |  | 6(50) | 35(51) | 1.00 |
| Caesarean section | 9(22) | 14(20) | 1.00 |  | 4(19) | 14(20) | 1.00 |  | 2(17) | 14(20) | 1.00 |
| Infants with older siblings | 23(56) | 32(46) | 0.43 |  | 14(67) | 32(46) | 0.14 |  | 9(75) | 32(46) | 0.16 |
| Exclusive Breast feeding | 24(59) | 45(65) | 0.54 |  | 12(57) | 45(65) | 0.61 |  | 9(75) | 45(65) | 0.74 |
| Age of Mothers | 30.7(5.4) | 31.9(4.6) | 0.25 |  | 31.7(4.9) | 31.9(4.6) | 0.73 |  | 30.0(6.2) | 31.9(4.6) | 0.35 |
| Antimicrobial exposure after birth from 6mo | 21(51) | 34(49) | 1.00 |  | 14(67) | 34(49) | 0.21 |  | 10(83) | 34(49) | 0.06 |
| Antimicrobial exposure after birth from 6mo to 12mo | 11(27) | 20(29) | 1.00 |  | 8(38) | 20(29) | 0.43 |  | 4(33) | 20(29) | 0.74 |

For each factor, the number of each category variable and the percentage it represents of the total are displayed as percentages, and for continuous variables, the mean and standard deviation are indicated. To compare the presence or absence of allergy development in each factor, the Mann-Whitney U test was used for continuous variables, and Pearson's chi-square test was used for categorical variables.

Supplementary Table S9 . The influence of background factors on the degree of dominance of each bacterial genus.

|  | *Bifidobacterium* | | |  | *Bacteroides* | | |  |  | *Clostridium* | | |  | *Faecalibacterium* |
| --- | --- | --- | --- | --- | --- | --- | --- | --- | --- | --- | --- | --- | --- | --- |
| **1month** | Odds Ratios | 95%CI |  |  | Odds Ratios | 95%CI |  |  |  | Odds Ratios | 95%CI |  |  | Odds Ratios |
| Infants without AD at 24 month | 1.924 | 0.825 | 4.484 |  | 2.295 | 0.916 | 5.75 |  |  | 1.275 | 0.432 | 3.765 |  | NA |
| Infants without RW at 24 month | 1.166 | 0.414 | 3.282 |  | 1.856 | 0.601 | 5.729 |  |  | 1.495 | 0.443 | 5.046 |  | NA |
| Infants without FA at 24 month | 0.903 | 0.225 | 3.619 |  | 0.999 | 0.214 | 4.663 |  |  | 2.184 | 0.287 | 16.607 |  | NA |
| Female | 0.922 | 0.411 | 2.067 |  | 1.137 | 0.466 | 2.774 |  |  | 0.609 | 0.234 | 1.584 |  | NA |
| Vaginal Delivery | 0.549 | 0.18 | 1.672 |  | >999.999 | <0.001 | >999.999 |  |  | 0.125 | 0.031 | 0.5 | ** | NA |
| Infants without siblings | 0.347 | 0.148 | 0.817 | * | 1.07 | 0.438 | 2.615 |  |  | 3.635 | 1.205 | 10.963 | * | NA |
| Non-AED | 2.828 | 1.114 | 7.177 | * | 1.348 | 0.545 | 3.336 |  |  | 0.235 | 0.08 | 0.694 | ** | NA |
| Exclusively breast-fed | 2.254 | 0.982 | 5.174 |  | 0.585 | 0.225 | 1.523 |  |  | 0.203 | 0.076 | 0.539 | ** | NA |
|  |  |  |  |  |  |  |  |  |  |  |  |  |  |  |
| **3month** | Odds Ratios | 95%CI |  |  | Odds Ratios | 95%CI |  |  |  | Odds Ratios | 95%CI |  |  | Odds Ratios |
| Infants without AD at 24 month | 2.085 | 0.91 | 4.777 |  | 1.798 | 0.756 | 4.278 |  |  | 0.45 | 0.151 | 1.344 |  | NA |
| Infants without RW at 24 month | 0.546 | 0.194 | 1.531 |  | 1.155 | 0.386 | 3.457 |  |  | 10.074 | 2.42 | 41.933 | ** | NA |
| Infants without FA at 24 month | 1.524 | 0.419 | 5.542 |  | 0.92 | 0.239 | 3.54 |  |  | 0.999 | 0.2 | 4.989 |  | NA |
| Female | 0.778 | 0.353 | 1.716 |  | 1.175 | 0.511 | 2.702 |  |  | 0.851 | 0.325 | 2.226 |  | NA |
| Vaginal Delivery | 0.732 | 0.247 | 2.171 |  | 15.279 | 3.029 | 77.06 | ** |  | 0.662 | 0.174 | 2.516 |  | NA |
| Infants without siblings | 0.308 | 0.132 | 0.717 | ** | 1.372 | 0.597 | 3.157 |  |  | 1.55 | 0.562 | 4.274 |  | NA |
| Non-AED | 3.001 | 1.191 | 7.562 | * | 1.414 | 0.584 | 3.425 |  |  | 0.098 | 0.033 | 0.289 | *** | NA |
| Exclusively breast-fed | 0.939 | 0.412 | 2.144 |  | 0.549 | 0.223 | 1.353 |  |  | 0.262 | 0.096 | 0.72 | ** | NA |
|  |  |  |  |  |  |  |  |  |  |  |  |  |  |  |
| **6month** | Odds Ratios | 95%CI |  |  | Odds Ratios | 95%CI |  |  |  | Odds Ratios | 95%CI |  |  | Odds Ratios |
| Infants without AD at 24 month | 1.017 | 0.467 | 2.213 |  | 0.911 | 0.412 | 2.017 |  |  | 0.577 | 0.239 | 1.389 |  | NA |
| Infants without RW at 24 month | 0.791 | 0.295 | 2.12 |  | 0.926 | 0.337 | 2.549 |  |  | 0.715 | 0.246 | 2.08 |  | NA |
| Infants without FA at 24 month | 1.621 | 0.442 | 5.942 |  | 0.602 | 0.166 | 2.178 |  |  | 1.726 | 0.426 | 6.984 |  | NA |
| Female | 0.506 | 0.238 | 1.077 |  | 2.018 | 0.933 | 4.367 |  |  | 1.59 | 0.711 | 3.554 |  | NA |
| Vaginal Delivery | 1.158 | 0.406 | 3.299 |  | 2.437 | 0.821 | 7.235 |  |  | 1.025 | 0.346 | 3.042 |  | NA |
| Infants without siblings | 0.75 | 0.346 | 1.624 |  | 0.584 | 0.263 | 1.294 |  |  | 0.567 | 0.246 | 1.306 |  | NA |
| Non-AED | 1.192 | 0.508 | 2.797 |  | 1.193 | 0.503 | 2.834 |  |  | 0.549 | 0.225 | 1.338 |  | NA |
| Exclusively breast-fed | 1.324 | 0.608 | 2.884 |  | 0.49 | 0.219 | 1.096 |  |  | 0.377 | 0.166 | 0.857 | * | NA |

|  | *Bifidobacterium* | | |  | *Bacteroides* | | |  |  | *Clostridium* | | |  | *Faecalibacterium* | | |
| --- | --- | --- | --- | --- | --- | --- | --- | --- | --- | --- | --- | --- | --- | --- | --- | --- |
| **9month** | Odds Ratios | 95%CI |  |  | Odds Ratios | 95%CI |  |  |  | Odds Ratios | 95%CI |  |  | Odds Ratios | 95%CI |  |
| Infants without AD at 24 month | 1.259 | 0.523 | 3.028 |  | 0.989 | 0.414 | 2.362 |  |  | 0.389 | 0.158 | 1.055 |  | NA |  |  |
| Infants without RW at 24 month | 0.678 | 0.231 | 1.99 |  | 2.109 | 0.723 | 6.148 |  |  | 1.11 | 0.393 | 3.135 |  | NA |  |  |
| Infants without FA at 24 month | 0.752 | 0.194 | 2.911 |  | 0.906 | 0.236 | 3.488 |  |  | 2.204 | 0.54 | 8.992 |  | NA |  |  |
| Female | 0.754 | 0.34 | 1.671 |  | 2.132 | 0.966 | 4.706 |  |  | 0.605 | 0.273 | 1.34 |  | NA |  |  |
| Vaginal Delivery | 0.565 | 0.186 | 1.712 |  | 5.252 | 1.646 | 16.76 | ** |  | 0.23 | 0.071 | 0.743 | * | NA |  |  |
| Infants without siblings | 1.111 | 0.487 | 2.534 |  | 0.832 | 0.366 | 1.889 |  |  | 0.967 | 0.423 | 2.211 |  | NA |  |  |
| Non-AED | 1.561 | 0.639 | 3.81 |  | 0.564 | 0.233 | 1.366 |  |  | 1.526 | 0.627 | 3.714 |  | NA |  |  |
| Exclusively breast-fed | 3.936 | 1.715 | 9.032 | ** | 1.003 | 0.446 | 2.256 |  |  | 0.594 | 0.263 | 1.343 |  | NA |  |  |
|  |  |  |  |  |  |  |  |  |  |  |  |  |  |  |  |  |
| **12month** | Odds Ratios | 95%CI |  |  | Odds Ratios | 95%CI |  |  |  | Odds Ratios | 95%CI |  |  | Odds Ratios | 95%CI |  |
| Infants without AD at 24 month | 0.771 | 0.313 | 1.902 |  | 0.905 | 0.352 | 2.324 |  |  | 1.155 | 0.474 | 2.813 |  | 0.991 | 0.382 | 2.571 |
| Infants without RW at 24 month | 0.325 | 0.101 | 1.045 |  | 3.964 | 1.147 | 13.696 | * |  | 0.483 | 0.162 | 1.443 |  | 0.875 | 0.291 | 2.63 |
| Infants without FA at 24 month | 1.412 | 0.328 | 6.08 |  | 0.854 | 0.193 | 3.776 |  |  | 1.405 | 0.334 | 5.919 |  | 2.733 | 0.498 | 14.991 |
| Female | 0.742 | 0.331 | 1.666 |  | 2.877 | 1.194 | 6.934 | * |  | 0.691 | 0.311 | 1.535 |  | 0.679 | 0.29 | 1.588 |
| Vaginal Delivery | 0.639 | 0.204 | 2.003 |  | 5.64 | 1.599 | 19.89 | ** |  | 0.507 | 0.168 | 1.532 |  | 0.67 | 0.21 | 2.138 |
| Infants without siblings | 0.799 | 0.346 | 1.845 |  | 1.343 | 0.563 | 3.201 |  |  | 0.977 | 0.42 | 2.272 |  | 0.554 | 0.225 | 1.364 |
| Non-AED | 0.729 | 0.287 | 1.851 |  | 0.474 | 0.175 | 1.282 |  |  | 2.532 | 0.981 | 6.534 |  | 1.025 | 0.374 | 2.809 |
| Exclusively breast-fed | 3.05 | 1.287 | 7.227 | * | 0.258 | 0.102 | 0.65 | ** |  | 0.368 | 0.157 | 0.862 | * | 0.944 | 0.391 | 2.278 |
| Without Abx exposure after 6mo to 12mo | 0.617 | 0.274 | 1.39 |  | 0.606 | 0.258 | 1.423 |  |  | 0.545 | 0.228 | 1.302 |  | 0.422 | 0.171 | 1.038 |
|  |  |  |  |  |  |  |  |  |  |  |  |  |  |  |  |  |
| **18month** | Odds Ratios | 95%CI |  |  | Odds Ratios | 95%CI |  |  |  | Odds Ratios | 95%CI |  |  | Odds Ratios | 95%CI |  |
| Infants without AD at 24 month | 0.855 | 0.345 | 2.119 |  | 0.988 | 0.392 | 2.49 |  |  | 0.902 | 0.377 | 2.157 |  | 1.518 | 0.615 | 3.745 |
| Infants without RW at 24 month | 0.497 | 0.154 | 1.609 |  | 5.135 | 1.377 | 19.155 | * |  | 0.674 | 0.221 | 2.056 |  | 0.57 | 0.181 | 1.798 |
| Infants without FA at 24 month | 0.183 | 0.033 | 1.012 |  | 1.974 | 0.431 | 9.031 |  |  | 0.7 | 0.187 | 2.631 |  | 0.799 | 0.206 | 3.09 |
| Female | 1.652 | 0.733 | 3.724 |  | 0.517 | 0.225 | 1.189 |  |  | 1.209 | 0.556 | 2.629 |  | 0.598 | 0.271 | 1.319 |
| Vaginal Delivery | 1.392 | 0.444 | 4.368 |  | 0.911 | 0.285 | 2.91 |  |  | 1.692 | 0.577 | 4.959 |  | 0.788 | 0.259 | 2.396 |
| Infants without siblings | 0.581 | 0.251 | 1.347 |  | 2.165 | 0.931 | 5.036 |  |  | 0.953 | 0.425 | 2.14 |  | 0.517 | 0.227 | 1.176 |
| Non-AED | 1.331 | 0.531 | 3.339 |  | 1.511 | 0.595 | 3.839 |  |  | 0.543 | 0.223 | 1.321 |  | 0.74 | 0.299 | 1.832 |
| Exclusively breast-fed | 1.549 | 0.671 | 3.576 |  | 0.571 | 0.241 | 1.351 |  |  | 0.702 | 0.314 | 1.572 |  | 1.329 | 0.582 | 3.034 |
| Without Abx exposure after 6mo to 12mo | 1.228 | 0.545 | 2.767 |  | 0.911 | 0.397 | 2.092 |  |  | 0.622 | 0.263 | 1.47 |  | 0.52 | 0.215 | 1.26 |
|  |  |  |  |  |  |  |  |  |  |  |  |  |  |  |  |  |
| **24month** | Odds Ratios | 95%CI |  |  | Odds Ratios | 95%CI |  |  |  | Odds Ratios | 95%CI |  |  | Odds Ratios | 95%CI |  |
| Infants without AD at 24 month | 0.249 | 0.089 | 0.697 | ** | 0.574 | 0.223 | 1.477 |  |  | 1.722 | 0.674 | 4.4 |  | 1.25 | 0.504 | 3.1 |
| Infants without RW at 24 month | 1.358 | 0.416 | 4.434 |  | 1.902 | 0.601 | 6.021 |  |  | 1.543 | 0.49 | 4.854 |  | 1.194 | 0.396 | 3.602 |
| Infants without FA at 24 month | 3.331 | 0.716 | 15.49 |  | 0.94 | 0.223 | 3.96 |  |  | 0.933 | 0.221 | 3.932 |  | 1.083 | 0.264 | 4.447 |
| Female | 1.617 | 0.715 | 3.66 |  | 0.729 | 0.33 | 1.61 |  |  | 0.631 | 0.285 | 1.397 |  | 0.985 | 0.454 | 2.136 |
| Vaginal Delivery | 1.736 | 0.543 | 5.555 |  | 1.374 | 0.439 | 4.298 |  |  | 2.486 | 0.786 | 7.861 |  | 1.17 | 0.386 | 3.548 |
| Infants without siblings | 0.444 | 0.188 | 1.049 |  | 1.182 | 0.516 | 2.704 |  |  | 0.555 | 0.243 | 1.268 |  | 1.085 | 0.486 | 2.424 |
| Non-AED | 1.584 | 0.607 | 4.13 |  | 0.462 | 0.183 | 1.166 |  |  | 0.471 | 0.185 | 1.202 |  | 0.686 | 0.277 | 1.698 |
| Exclusively breast-fed | 1.166 | 0.493 | 2.762 |  | 0.528 | 0.228 | 1.224 |  |  | 1.204 | 0.515 | 2.815 |  | 1.166 | 0.512 | 2.655 |
| Without Abx exposure after 6m.o. | 0.873 | 0.307 | 2.481 |  | 0.849 | 0.305 | 2.363 |  |  | 1.335 | 0.483 | 3.69 |  | 0.953 | 0.353 | 2.571 |
| Without Abx exposure after 6mo to 12mo | 1.893 | 0.608 | 5.892 |  | 0.731 | 0.244 | 2.192 |  |  | 0.925 | 0.309 | 2.767 |  | 0.482 | 0.164 | 1.418 |

To investigate the relationship between each background factor and the occupancy of each bacterial species, the occupancy of each bacterial species was divided into two groups by the median, and the odds ratio and 95% confidence interval were calculated by logistic regression analysis. The significance level was set at 5%.. *p < 0.05, **p < 0.01, ***p < 0.001.　AED means infants group with antimicrobial exposure at delivery. AD, RW, FA refer to groups of infants with atopic dermatitis, recurrent wheezing, and food allergy, respectively. NA represents those for which occupancy was not analyzed for comparison due to the large number of individuals with zero occupancy in either group.

Supplementary Table S10 . Comparison of diversity by presence or absence of allergy development in all age groups (all analysis target populations and each subgroup).

| Atopic dermatitis | Non-Allergy(N=69) vs AD(N=41) | 1m.o. | 3 m.o. | 6 m.o. | 9 m.o. | 12 m.o. | 18 m.o. | 24 m.o. |
| --- | --- | --- | --- | --- | --- | --- | --- | --- |
| αdiversity | Chao1 | 0.11 | 0.93 | 0.54 | 0.57 | 0.3 | 0.88 | 0.84 |
|  | Shannon | 0.17 | 0.6 | 0.15 | 0.1 | 0.13 | 0.68 | 0.18 |
| βdiversity | Unweighted | 0.7 | 0.33 | 0.27 | 0.12 | 0.98 | 0.44 | 0.65 |
|  | Weighted | 0.07 | 0.46 | 0.34 | 0.02 | 0.57 | 0.2 | 0.17 |
|  |  |  |  |  |  |  |  |  |
| Recurrent wheezing | Non-Allergy(N=69) vs RW(N=21) | 1m.o. | 3 m.o. | 6 m.o. | 9 m.o. | 12 m.o. | 18 m.o. | 24 m.o. |
| αdiversity | Chao1 | 0.56 | 0.3 | 0.27 | 0.8 | 0.45 | 0.73 | 0.82 |
|  | Shannon | 0.22 | 0.89 | 0.03 | 0.54 | 0.15 | 0.55 | 0.58 |
| βdiversity | Unweighted | 0.56 | 0.68 | 0.38 | 0.9 | 0.86 | 0.41 | 0.55 |
|  | Weighted | 0.78 | 0.28 | 0.54 | 0.06 | 0.7 | 0.03 | 0.79 |
|  |  |  |  |  |  |  |  |  |
| Food allergy | Non-Allergy(N=69) vs FA(N=12) | 1m.o. | 3 m.o. | 6 m.o. | 9 m.o. | 12 m.o. | 18 m.o. | 24 m.o. |
| αdiversity | Chao1 | 0.88 | 0.41 | 0.91 | 0.59 | 0.73 | 0.38 | 0.84 |
|  | Shannon | 0.46 | 0.78 | 0.03 | 0.03 | 0.43 | 0.39 | 0.51 |
| βdiversity | Unweighted | 0.98 | 0.82 | 0.13 | 0.7 | 0.91 | 0.23 | 0.27 |
|  | Weighted | 0.71 | 0.56 | 0.31 | 0.15 | 0.3 | 0.12 | 0.07 |

The p-values for the results of the Shannon and Chao1 analyses for alpha diversity and the weighted and unweighted analyses for beta diversity are listed for each month of age for the entire analysis population and each subgroup, comparing the results for the allergy-onset group and non-onset group. AED and Non-AED indicate infants with and without antimicrobial exposure at delivery, respectively. Sib and Non-Sib indicate infants with and without older siblings, respectively.

Supplementary Figure1. Background factors affecting the relative abundance of *Bacterroides, Clostridium, and Faecalibacterium.*


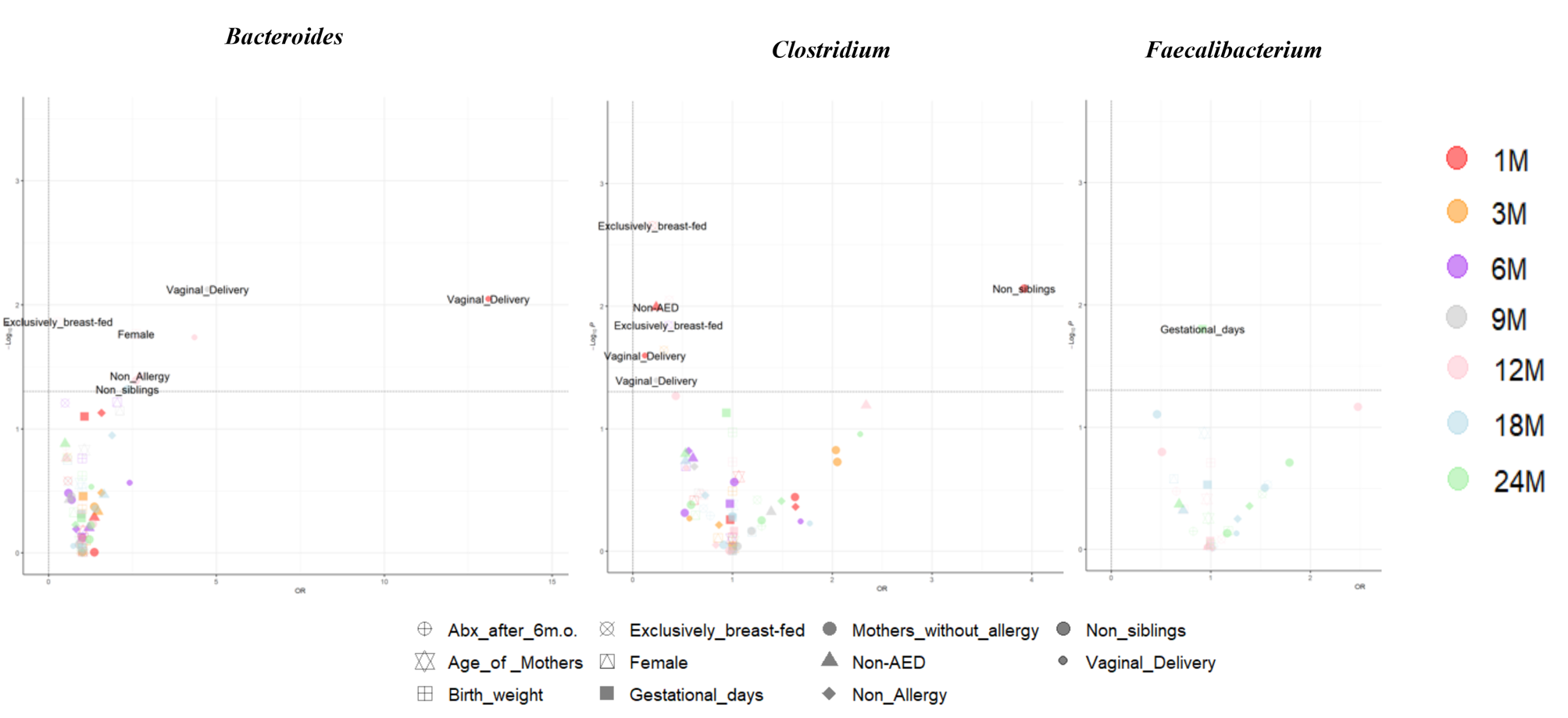


To visualise the influence of background factors on the relative abundance of *Bacterroides, Clostridium, and Faecalibacterium*, two values representing low and high relative abundances were selected based on the median, and volcano plots were created with the odds ratio for the direction of high relative abundance on the x-axis and the p-value logarithm (-Log_10_P) on the y-axis. Each factor is colour-coded according to the age of the subject. Abx_after_6m.o. indicates exposure to antibiotics after 6 months of age, and AED denotes Antimicrobial Exposure at Delivery.

Supplementary Figure S2 .


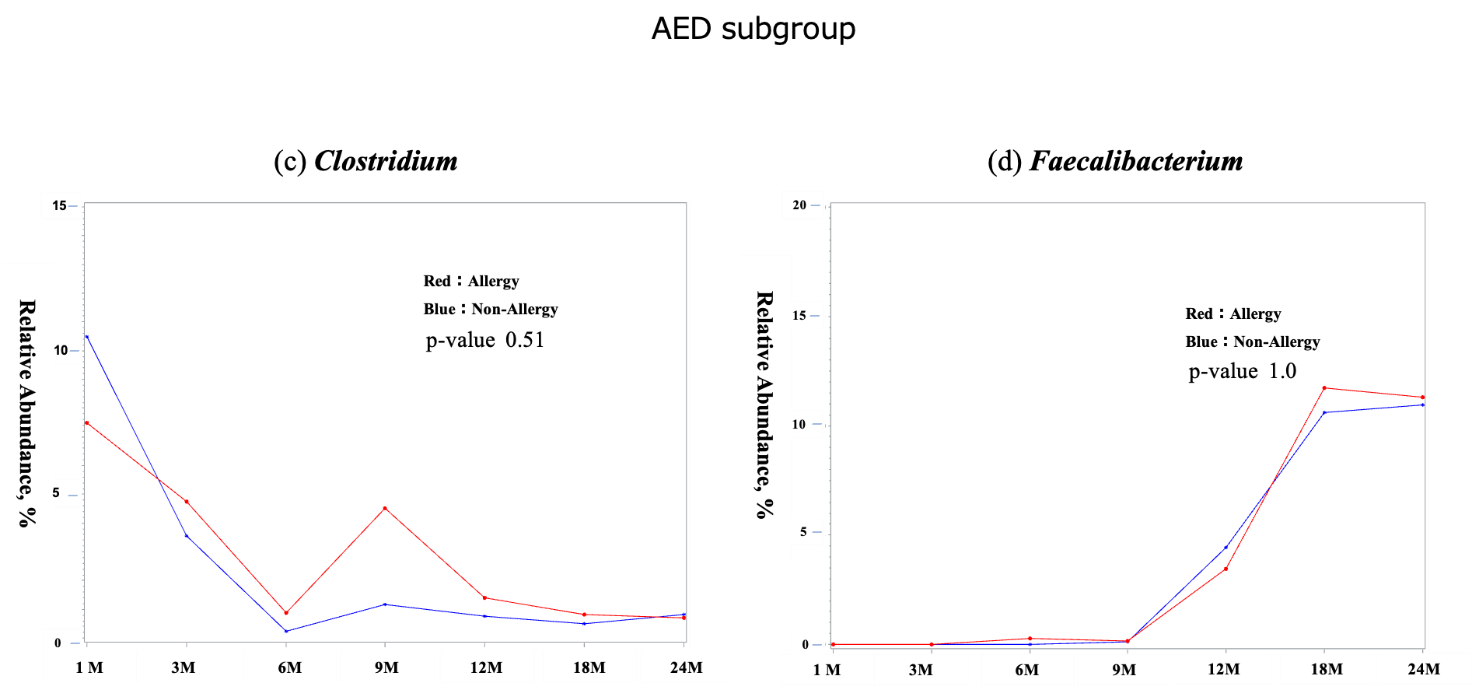


Comparison of the occupancies of genera, between infants at each month age with and without allergies by the age of 24 months in a subgroup of infants with antimicrobial exposure at delivery (total analyzed number was 66). In each graph for each bacterial genus, the vertical axis shows the relative abundance in the intestinal environment, and the horizontal axis shows the seven time points from birth to 24 months (1 month, 3 months, 6 months, 9 months, 12 months, 18 months, 24 months). The comparison of changes in the occupancy rate over time using a linear mixed-effects model is shown by the red line for the group that developed allergies by 24 months of age and the blue line for the non-allergic group, and the p-value is shown as the result of the comparison. In addition, for the comparison of occupancy rates (Mann-Whitney‘s U test was used) at each time point, the standard error of the occupancy rate is shown as a bar for those that showed a significant difference.　The significance level was set at 5%. *p < 0.05, **p < 0.01, ***p < 0.001.

Supplementary Figure S3 .


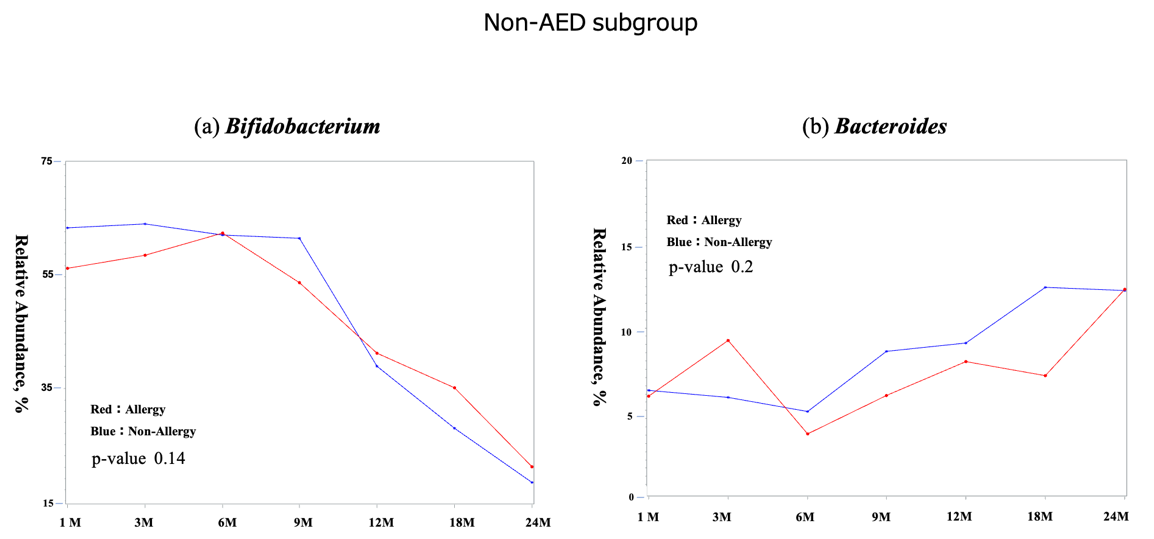


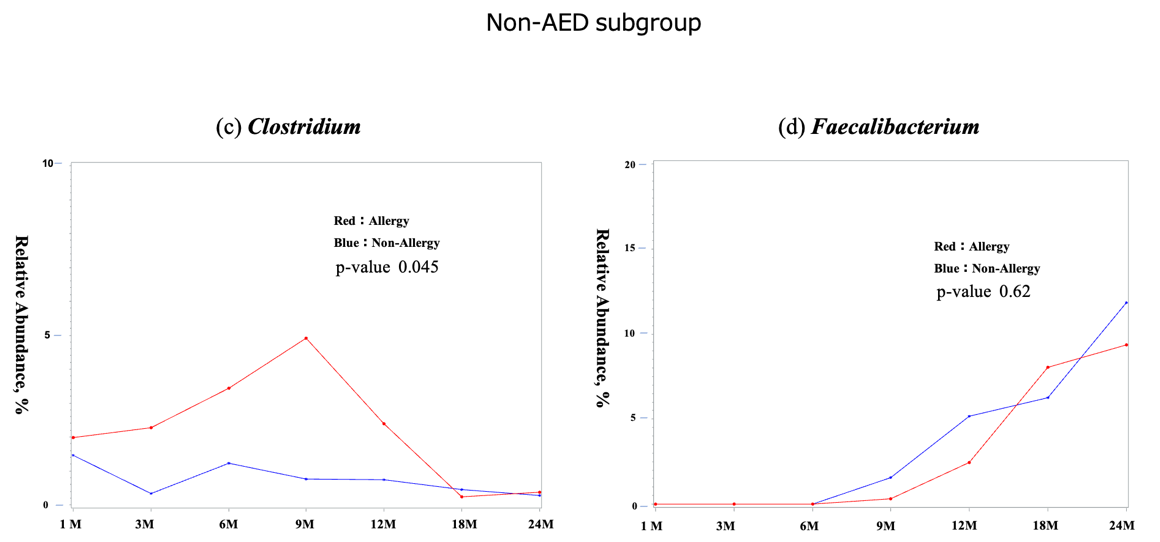


Comparison of the occupancies of genera, between infants at each month age with and without allergies by the age of 24 months in a subgroup of infants without antimicrobial exposure at delivery (total analyzed number was 55). The results of the diversity analysis are only shown for the months in which a significant difference was found, and a p-value indicates each result. In each graph for each bacterial genus, the vertical axis shows the relative abundance in the intestinal environment, and the horizontal axis shows the seven time points from birth to 24 months (1 month, 3 months, 6 months, 9 months, 12 months, 18 months, 24 months). The comparison of changes in the occupancy rate over time using a linear mixed-effects model is shown by the red line for the group that developed allergies by 24 months of age and the blue line for the non-allergic group, and the p-value is shown as the result of the comparison.

Supplementary Figure S4.


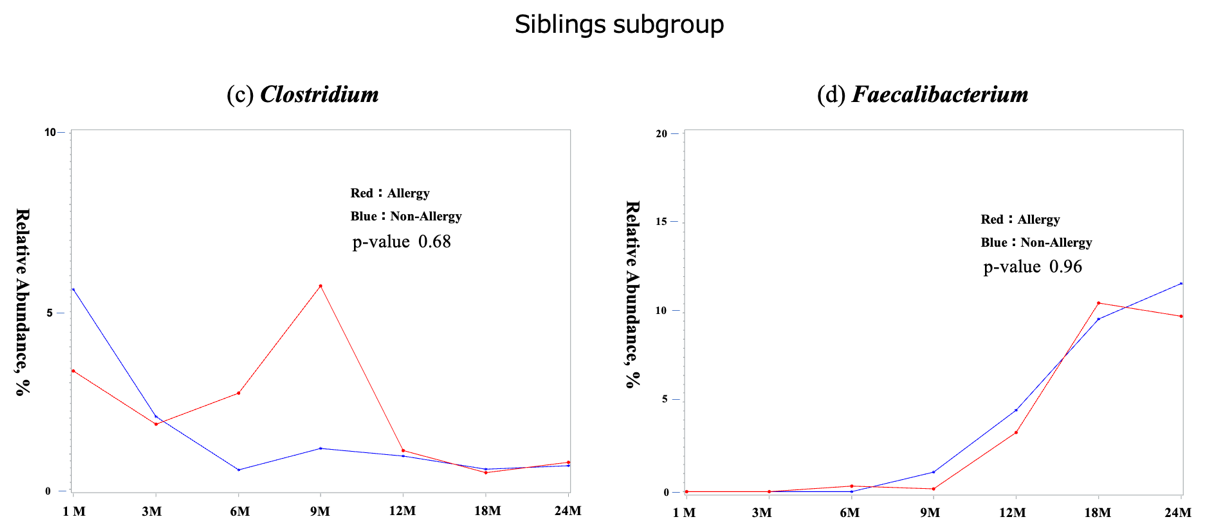


Comparison of the occupancy of each bacterial genus between infants at each month age with and without allergies by the age of 24 months in a subgroup of infants with older siblings (total analyzed number was 62). In each graph for each bacterial genus, the vertical axis shows the relative abundance in the intestinal environment, and the horizontal axis shows the seven time points from birth to 24 months (1 month, 3 months, 6 months, 9 months, 12 months, 18 months, 24 months). The comparison of changes in the occupancy rate over time using a linear mixed-effects model is shown by the red line for the group that developed allergies by 24 months of age and the blue line for the non-allergic group, and the p-value is shown as the result of the comparison.

Supplementary Figure S5 .


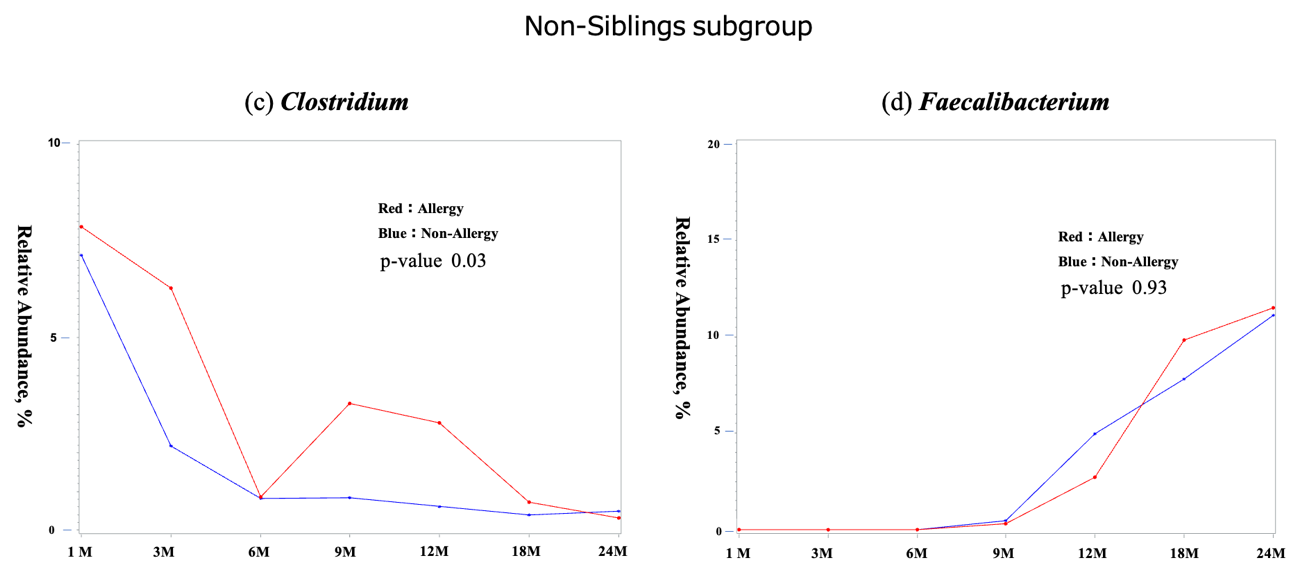


Comparison of the occupancies of genera, between infants at each month age with and without allergies by the age of 24 months in a subgroup of infants without older siblings (total analyzed number was 59). In each graph for each bacterial genus, the vertical axis shows the relative abundance in the intestinal environment, and the horizontal axis shows the seven time points from birth to 24 months (1 month, 3 months, 6 months, 9 months, 12 months, 18 months, 24 months). The comparison of changes in the occupancy rate over time using a linear mixed-effects model is shown by the red line for the group that developed allergies by 24 months of age and the blue line for the non-allergic group, and the p-value is shown as the result of the comparison.

Supplementary Figure S6 .


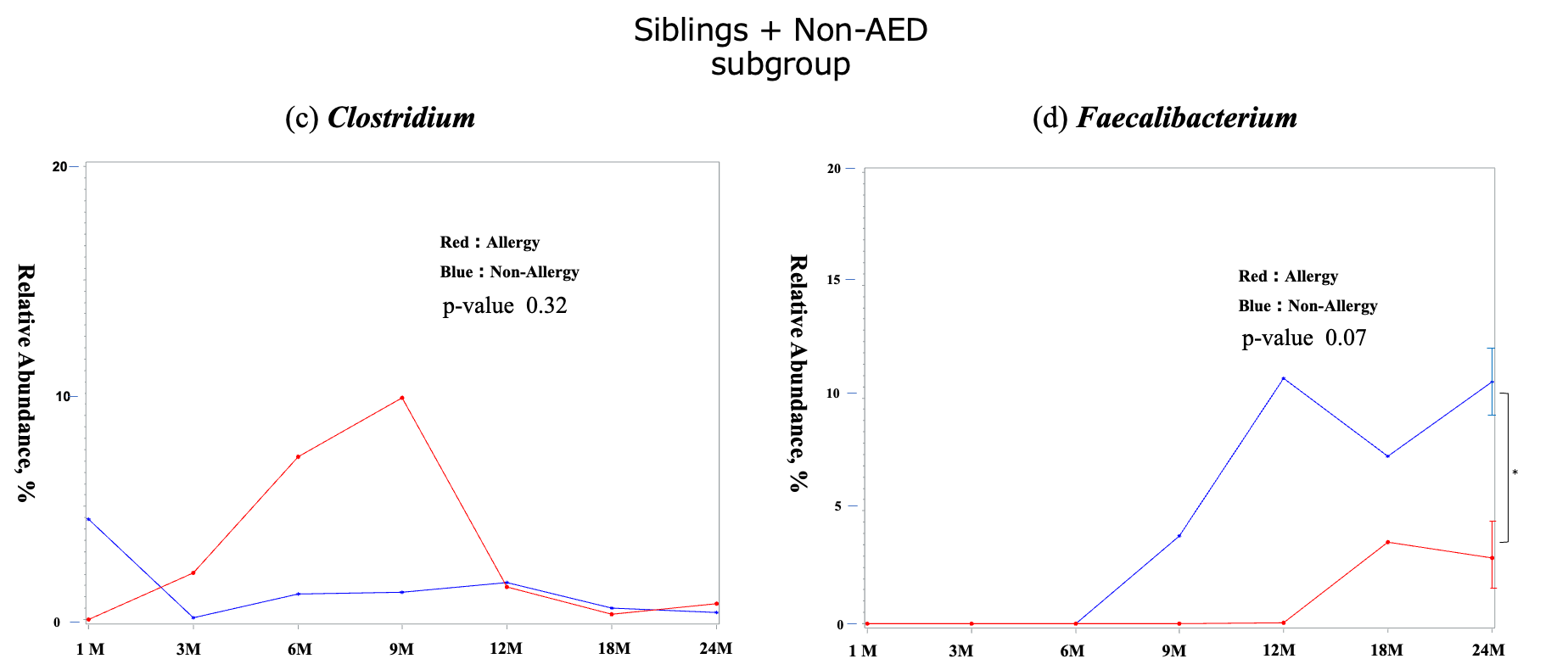


Comparison of the occupancies of genera, between infants at each month age with and without allergies by the age of 24 months in a subgroup of infants without antimicrobial exposure and with older siblings (total analyzed number was 22). In each graph for each bacterial genus, the vertical axis shows the relative abundance in the intestinal environment, and the horizontal axis shows the seven time points from birth to 24 months (1 month, 3 months, 6 months, 9 months, 12 months, 18 months, 24 months). The comparison of changes in the occupancy rate over time using a linear mixed-effects model is shown by the red line for the group that developed allergies by 24 months of age and the blue line for the non-allergic group, and the p-value is shown as the result of the comparison. In addition, for the comparison of occupancy rates (Mann-Whitney‘s U test was used) at each time point, the standard error is shown as a bar for those that showed a significant difference.　The significance level was set at 5%. *p < 0.05, **p < 0.01, ***p < 0.001.

Supplementary Figure S7 .


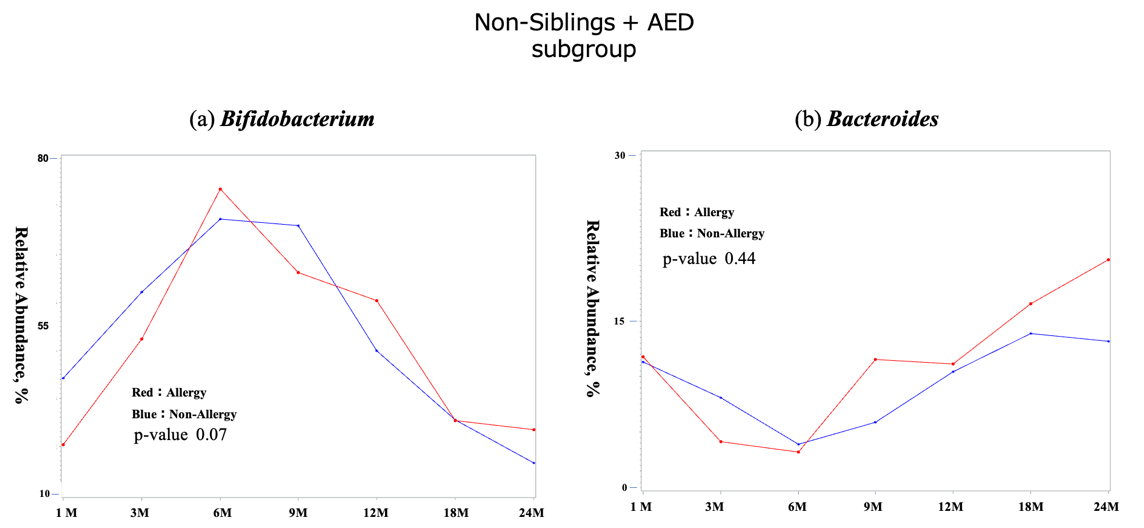


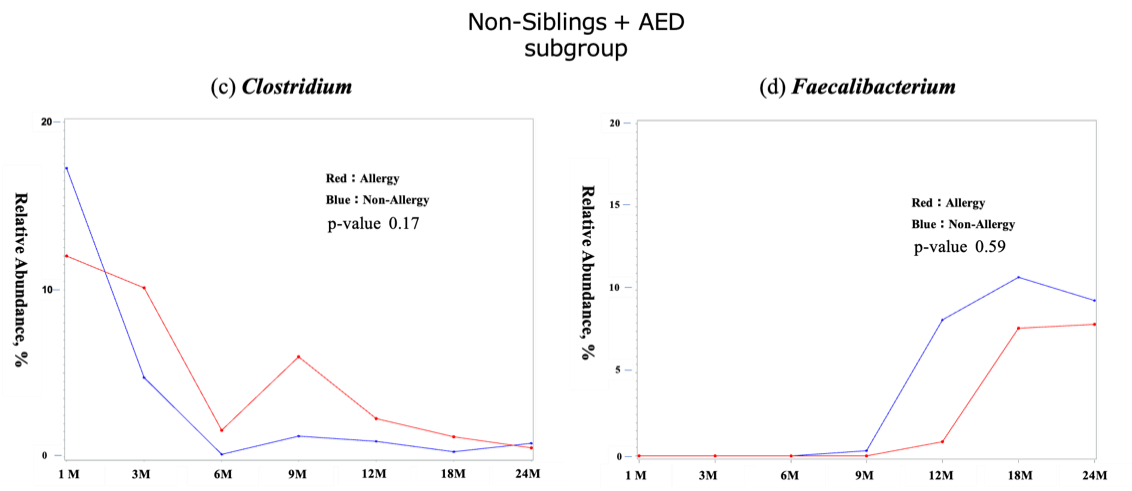


Comparison of the occupancies of genera, between infants at each month age with and without allergies by the age of 24 months in a subgroup of infants with antimicrobial exposure and without older siblings (total analyzed number was 26). In each graph for each bacterial genus, the vertical axis shows the relative abundance in the intestinal environment, and the horizontal axis shows the seven time points from birth to 24 months (1 month, 3 months, 6 months, 9 months, 12 months, 18 months, 24 months). The comparison of changes in the occupancy rate over time using a linear mixed-effects model is shown by the red line for the group that developed allergies by 24 months of age and the blue line for the non-allergic group, and the p-value is shown as the result of the comparison.

Supplementary Figure S8 .


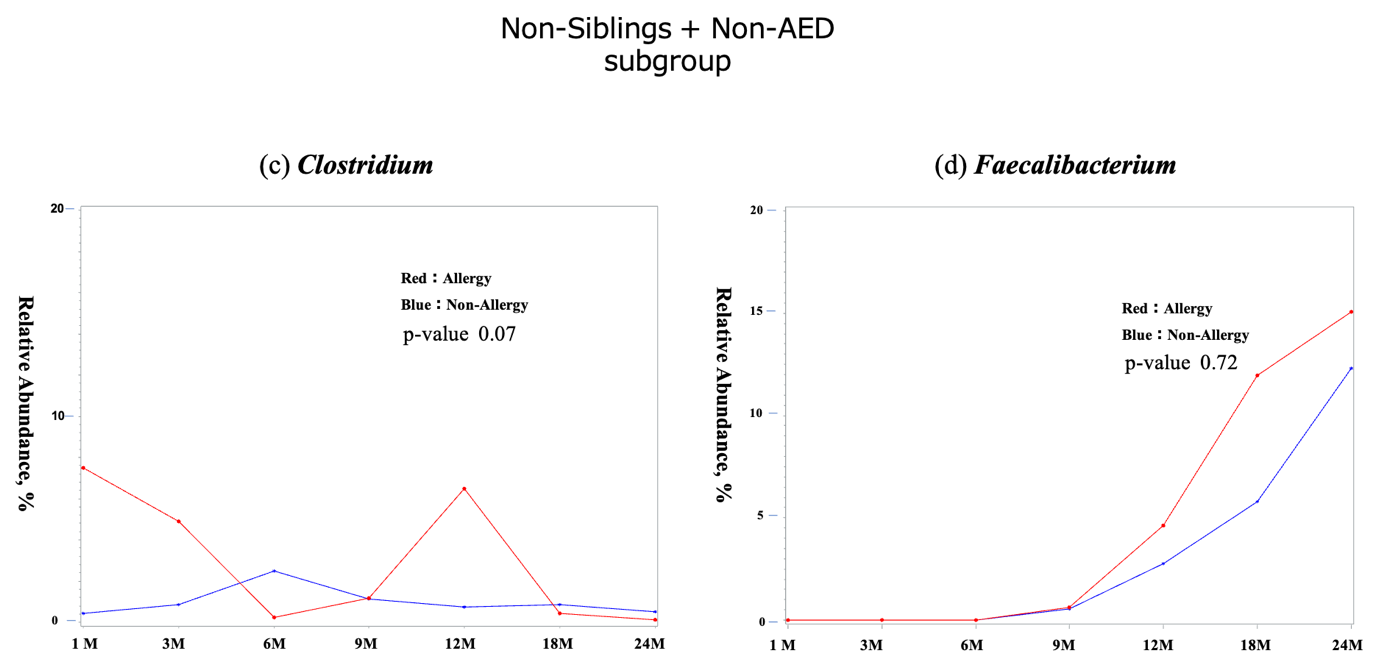


Comparison of the occupancies of genera, between infants at each month age with and without allergies by the age of 24 months in a subgroup of infants without antimicrobial exposure and without older siblings (total analyzed number was 33). In each graph for each bacterial genus, the vertical axis shows the relative abundance in the intestinal environment, and the horizontal axis shows the seven time points from birth to 24 months (1 month, 3 months, 6 months, 9 months, 12 months, 18 months, 24 months). The comparison of changes in the occupancy rate over time using a linear mixed-effects model is shown by the red line for the group that developed allergies by 24 months of age and the blue line for the non-allergic group, and the p-value is shown as the result of the comparison.

Supplementary Figure S8 .


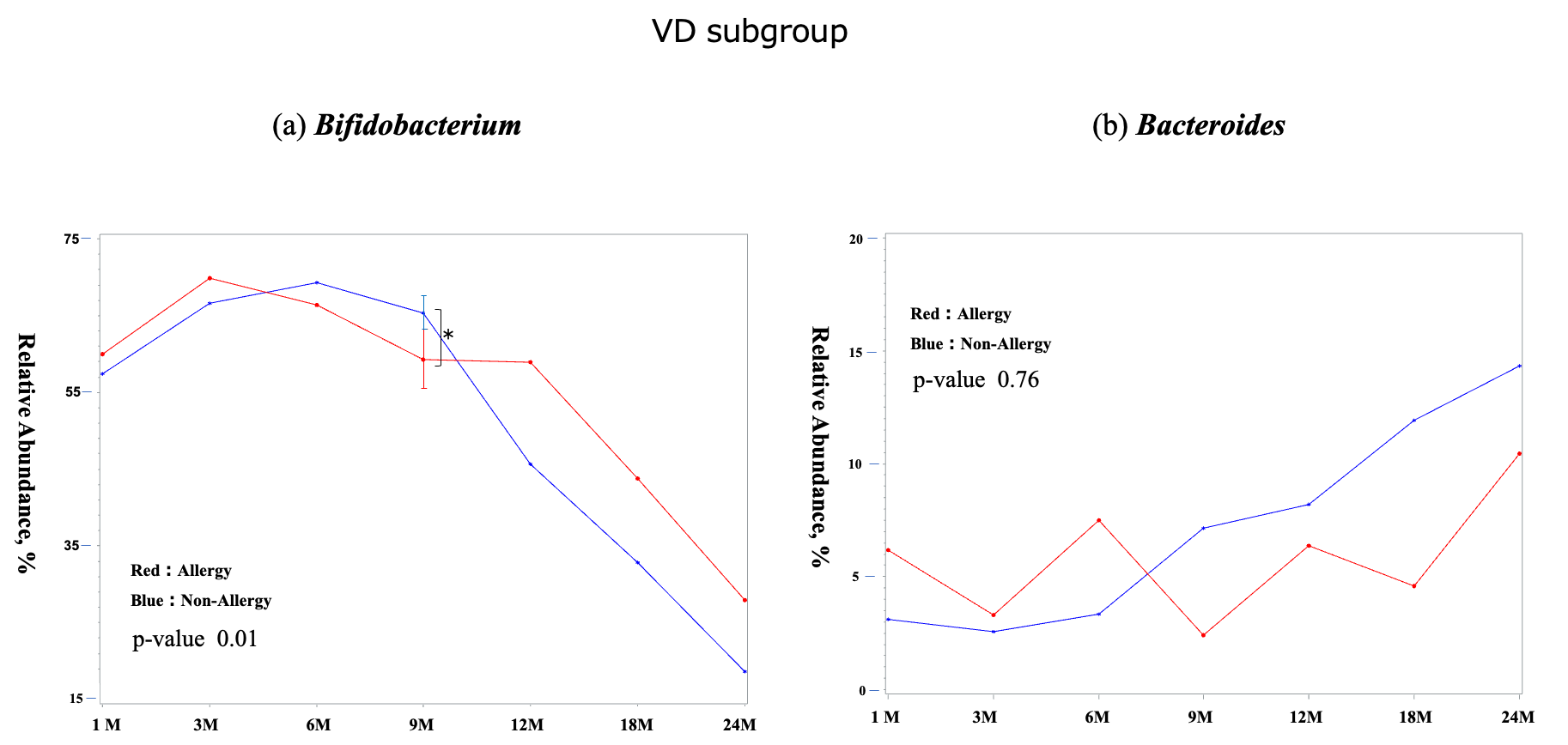


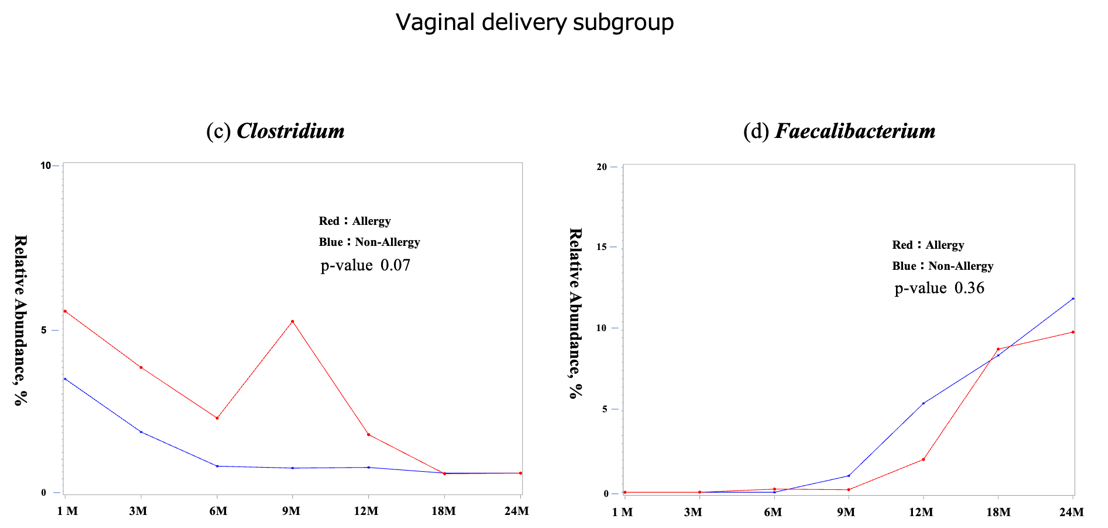


Comparison of the occupancies of genera, between infants at each month age with and without allergies by the age of 24 months in a subgroup of infants born by vaginal delivery (total analyzed number was 96). In each graph for each bacterial genus, the vertical axis shows the relative abundance in the intestinal environment, and the horizontal axis shows the seven time points from birth to 24 months (1 month, 3 months, 6 months, 9 months, 12 months, 18 months, 24 months). The comparison of changes in the occupancy rate over time using a linear mixed-effects model is shown by the red line for the group that developed allergies by 24 months of age and the blue line for the non-allergic group, and the p-value is shown as the result of the comparison. In addition, for the comparison of occupancy rates (Mann-Whitney‘s U test was used) at each time point, the standard error is shown as a bar for those that showed a significant difference.　The significance level was set at 5%. *p < 0.05, **p < 0.01, ***p < 0.001.

Supplementary Figure S10 .


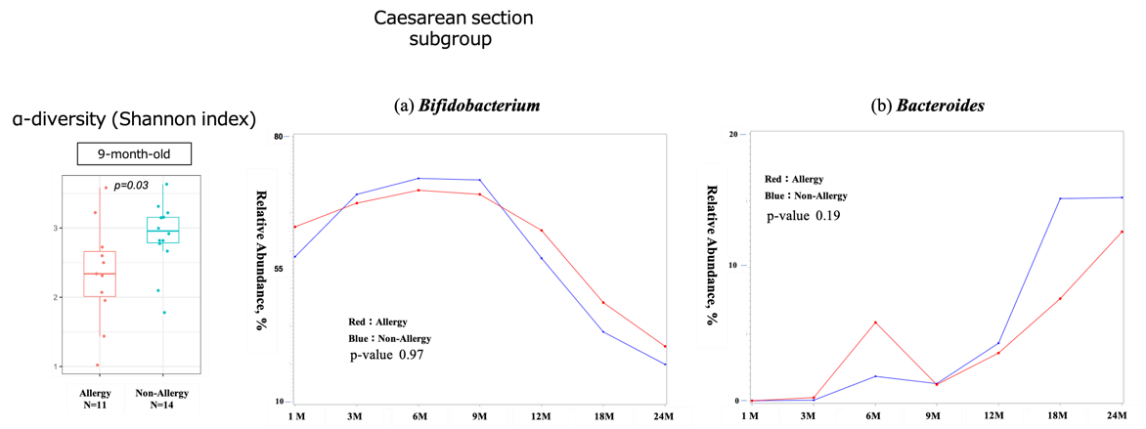


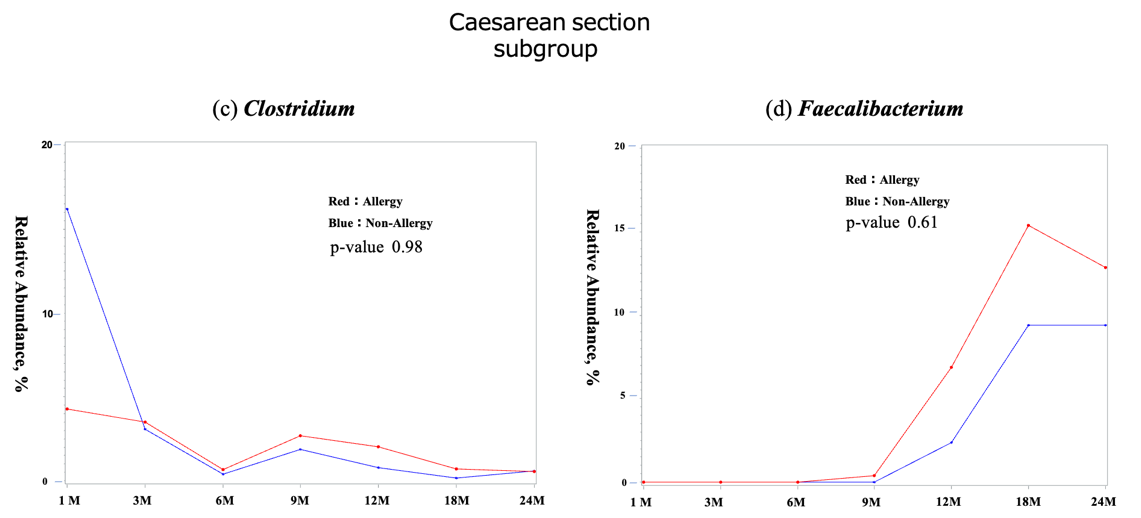


Comparison of diversity of gut environment, and the occupancies of genera, between infants at each month age with and without allergies by the age of 24 months in a subgroup of infants born by caesarean section (total analyzed number was 25). The results of the diversity analysis are only shown for the months in which a significant difference was found, and a p-value indicates each result. In each graph for each bacterial genus, the vertical axis shows the relative abundance in the intestinal environment, and the horizontal axis shows the seven time points from birth to 24 months (1 month, 3 months, 6 months, 9 months, 12 months, 18 months, 24 months). The comparison of changes in the occupancy rate over time using a linear mixed-effects model is shown by the red line for the group that developed allergies by 24 months of age and the blue line for the non-allergic group, and the p-value is shown as the result of the comparison.

Supplementary Figure S11 .


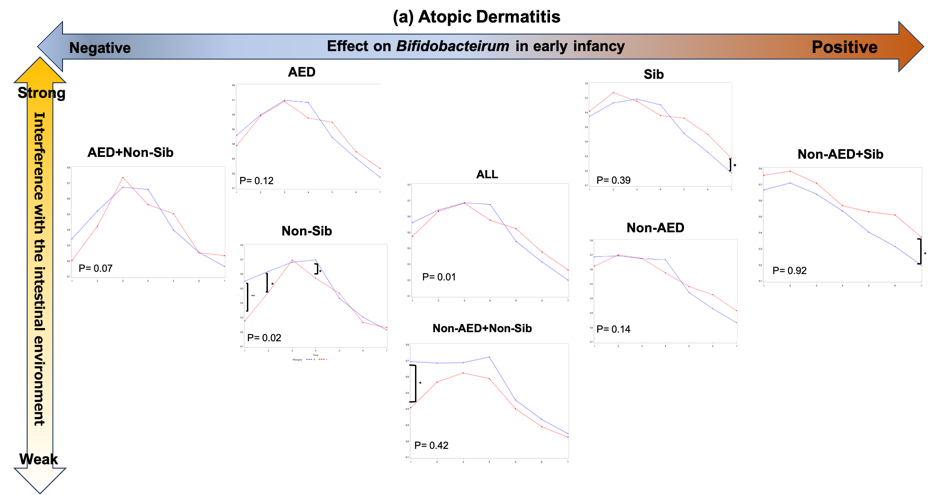

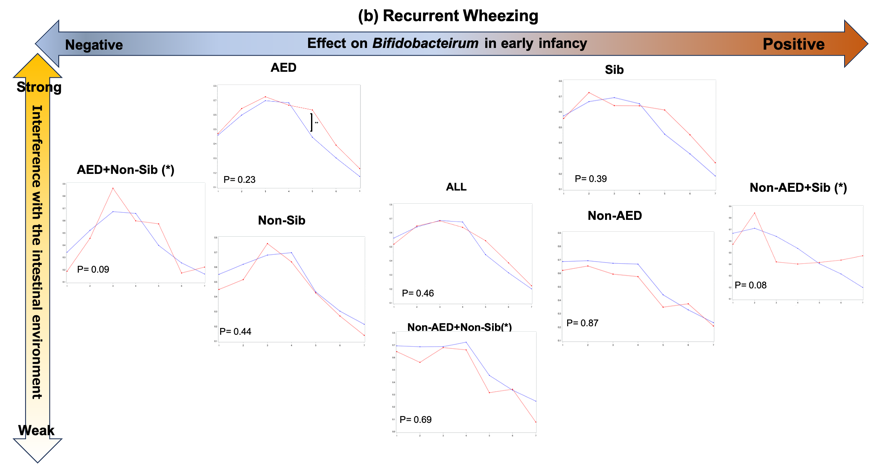


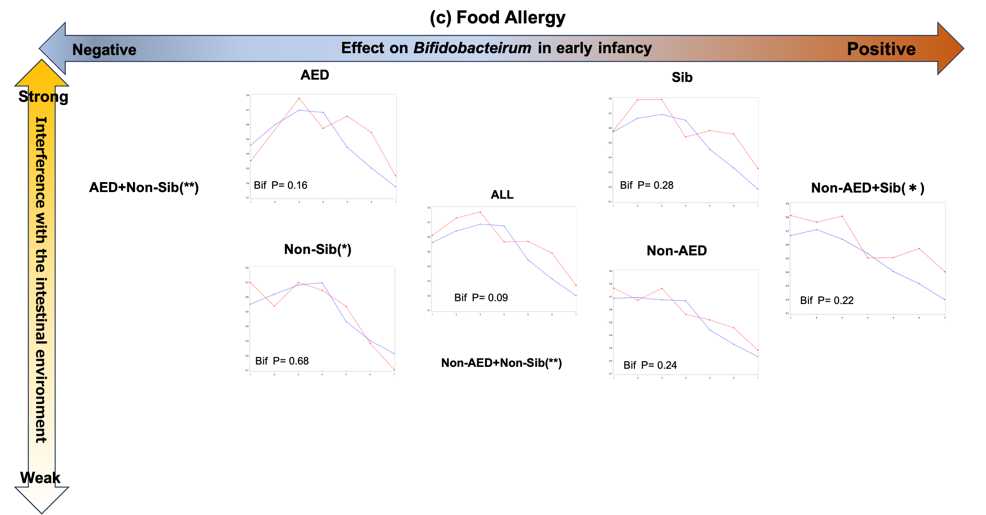


(a) Comparison of the occupancy of Bifidobacterium genus and its change over time in each group (including the all analysis target population and each subgroup) with and without the development of atopic dermatitis by 24 months age, depending on the direction of the effect on the intestinal flora in the early stages of infancy and the magnitude of interference. ALL indicates all analysis target population. AED and Non-AED indicate infants with and without antimicrobial exposure at delivery, respectively. Sib and Non-Sib indicate infants with and without older siblings, respectively. In the graphs for each group, the vertical axis shows the relative abundance of Bifidobacterium in the intestinal environment, and the horizontal axis shows the age of the infant (1 month, 3 months, 6 months, 9 months, 12 months, 18 months, 24 months) from youngest to oldest, numbered 1 to 7. The red line represents the relative values of the allergy onset group, and the blue line represents the relative values of the non-onset group, showing the change over time. The p-value in the graph shows the results of comparing the change over time in the two groups using a linear mixed effects model. The results of the comparison of the proportion of each group at each age (Mann-Whitney's U test) are also shown in each graph for those that showed a significant difference. The significance level was set at less than 5%. *p < 0.05, ** p< 0.01.
